# Supplementary material for: Remote near infrared identification of pathogens with multiplexed nanosensors
Source: Nat Commun. 2020 Nov 25;11:5995. doi: 10.1038/s41467-020-19718-5 (PMC7689463; doi:10.1038/s41467-020-19718-5)
Supplement: Supplementary file 2 — Supplementary Information [file 41467_2020_19718_MOESM2_ESM.pdf]

Supplementary Information

Remote near infrared identification of pathogens with multiplexed nanosensors

Robert Nißler,[1,5] *Oliver Bader,*[2] *Maria Dohmen,*[1] *Sebastian G. Walter,*[3] *Christine Noll,*[2] *Gabriele Selvaggio,*[1,5] *Uwe Groß,*[2] *Sebastian Kruss*[1,4,5*]

[1]Institute of Physical Chemistry, Göttingen University, Germany

[2]Institute of Medical Microbiology, Göttingen University Medical Center, Germany

[3]Department for Cardiothoracic Surgery and Intensive Care, University Hospital Cologne, Cologne, Germany

[4]Fraunhofer Institute for Microelectronic Circuits and Systems, Germany

[5]Physical Chemistry II, Bochum University, Germany

* corresponding author: Sebastian Kruss (sebastian.kruss@rub.de)

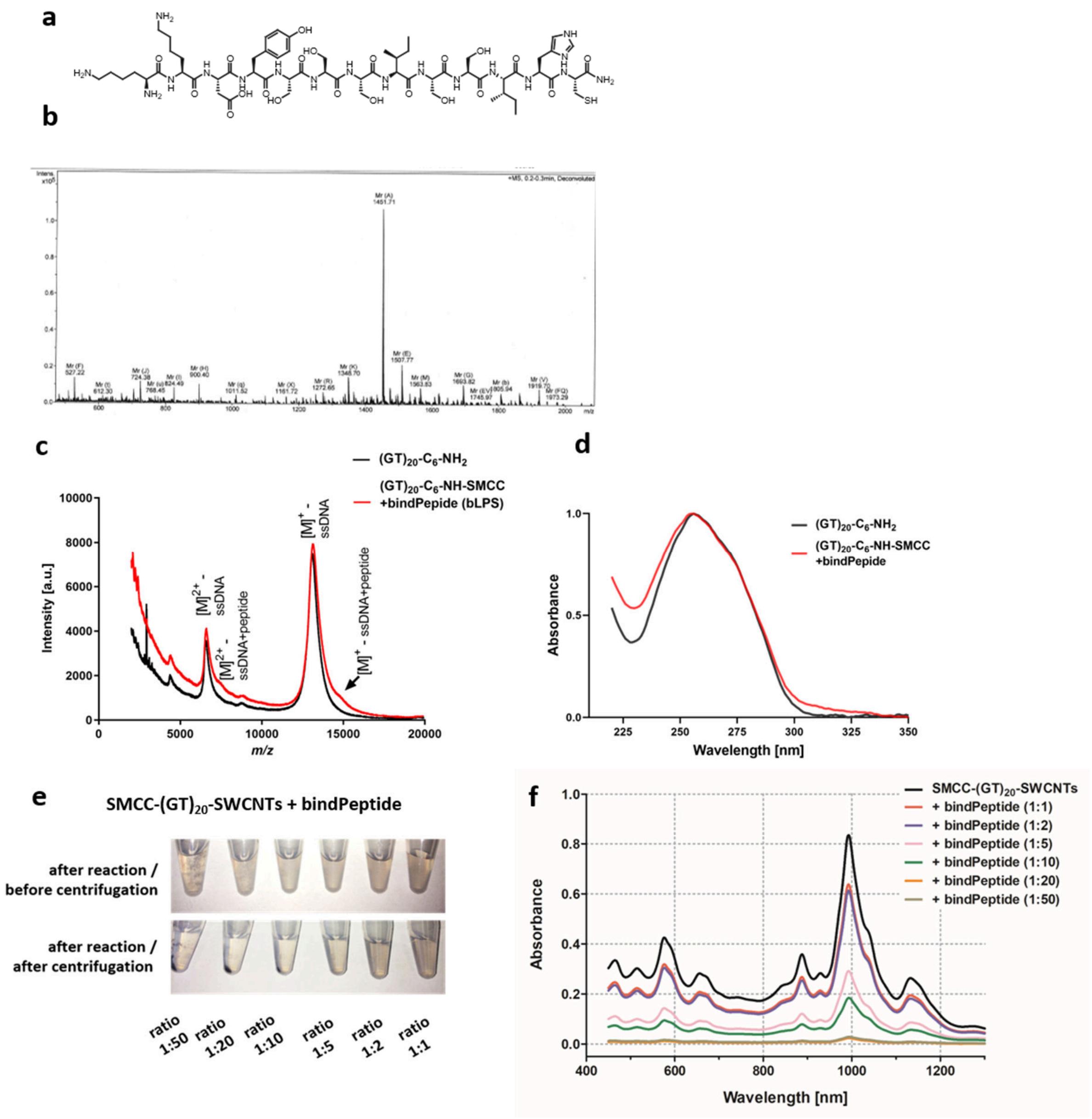

Figure S1: Lipopolysaccharide (LPS) sensor development.

a) Chemical formula and b) mass spectroscopy (MS) analysis of the synthesized LPS-binding peptide (detected: 1451.71 Da, expected 1452.64 Da). MS was acquired in positive mode, using direct injection and electron spray ionization with an microOTOF (Bruker Daltonics) device. c) Matrix-assisted Laser Desorption/Ionization (MALDI)-MS spectra of the single stranded (ss)DNA before and after conjugation with the (linked) LPS-binding peptide, acquired with Autoflex Speed (MALDI-TOF, Bruker Daltonics) in positive mode with 3-hydroxypicolinic acid matrix. The mean spectra from each 10 different spots on the MALDI target plate, show a ~1550 Da shifted [M]+ peak, which corresponds well with the additional mass from the peptide linked to SMCC (~1700 Da). This indicates a successful coupling of the LPS-binding peptide to ssDNA, which is in combination with a SWCNT the building block of the bLPS-sensor. The peak ratios should not be quantitatively interpreted as ionization and detection were optimized for ssDNA ($(GT)_6$-$(GT)_{30}$). d) Normalized UV absorbance spectra (Nanodrop 2000c, Thermo Scientific) show a shift towards peptide absorbance features at ~230 nm after peptide conjugation. e) Screening for optimal conjugation conditions regarding the SMCC-linked-$(GT)_{20}$-SWCNTs and the LPS-binding peptide. Different ratios of SMCC-DNA-SWCNTs to peptide were used, showing that for ratios above 1:5, the nanoconjugates form visible aggregates after overnight reaction and are no longer colloidally stable. f) Corresponding absorbance spectra for different reaction conditions show that for 1:1 and 1:2 ratios E_{11} absorbance of (6,5)-SWCNTs (at 992.2 nm) only slightly decreases. No wavelength shift was observed even for higher ratios. Based on these results, a ratio of 1:1 to 1:2 was used as final conjugation parameters.

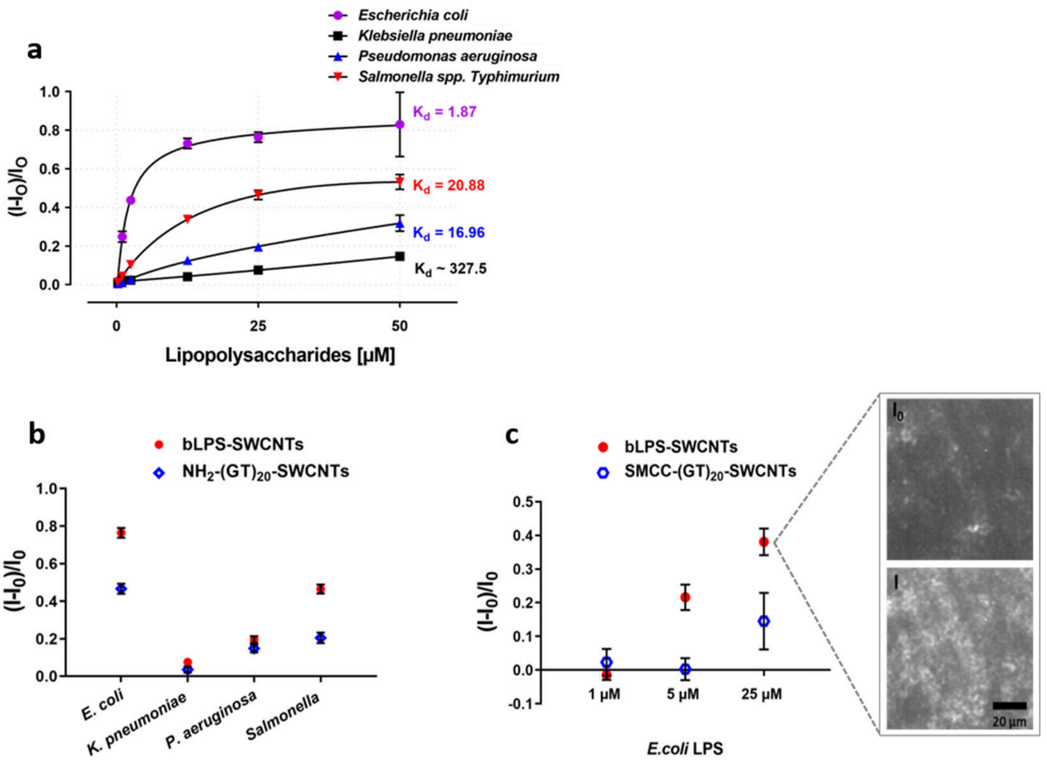

Figure S2: LPS sensor characterization.

a) Calibration curve of bLPS-SWCNT for LPS from different bacteria. *Escherichia coli* LPS showed the strongest response and the lowest K_d = 1.87 µM (Binding fit, nonlinear curve fit (GraphPad Prism 8), data from Fig. 2c) (n = 3 independent experiments, mean ± SD). b) Fluorescence response to 25 µM LPS for NH_2-(GT)$_{20}$-SWCNTs (blue) and bLPS-SWCNTs (red). The results show a non-specific smaller response of ssDNA-SWCNT to LPS probably due to sugar moieties in LPS (n = 3 independent experiments, mean ± SD). c) Fluorescence response of nanosensors immobilized on a glass surface. bLPS-SWCNTs (red) increase their fluorescence in response to 5 – 25 µM *E. coli* LPS, which is significantly higher than the unspecific response for sensors without the LPS-binding peptide (blue) (n = 3 independent experiments, mean ± SD). Therefore, the conjugation of the LPS-binding unit enhances sensing of the bacterial target.

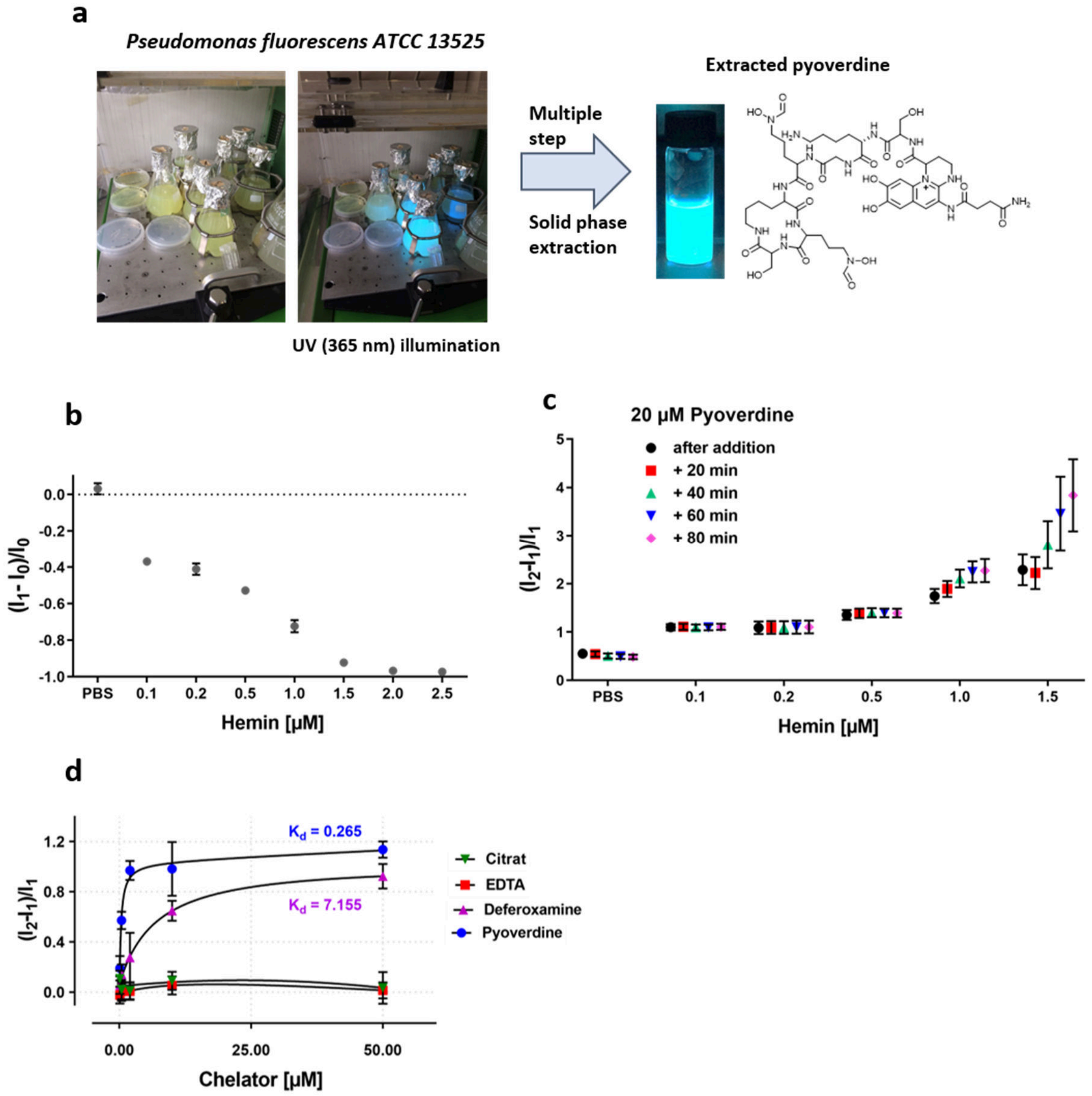

Figure S3: Siderophore sensing with hemin-aptamer-(HeApta)-SWCNTs.

a) Illustration of the pyoverdine extraction from *Pseudomonas fluorescens* ATCC 13525: Photographs of bacterial cultures with UV illumination show blue fluorescence of pyoverdines. A cascade of solid phase extraction (SPE) with fractionated elution from a C18ec resin isolates pyoverdines. b) Addition of hemin to HeApta-SWCNTs quenches the fluorescence emission (I_1) in a concentration dependent manner. 2 µM and more lead to complete quenching (n = 3 independent experiments, mean ± SD). c) Addition of 20 µM pyoverdine leads to a fluorescence increase (I_2), which correlates with the previous quenching (n = 3 independent experiments, mean ± SD). This indicates that dequenching of HeApta-SWCNTs (with hemin) in response to pyoverdine can be attributed to countering iron chelation. For further experiments, a hemin concentration of 1 µM was used for HeAtpa-SWCNTs (0.1 absorption at 993 nm) quenching, as it shows the highest relative fluorescence increase, without leading to non-fluorescent or non-stable sensors. Previous studies from Wu and Nißler *et al.* [1] showed furthermore that (6,5)-chirality enriched SWCNTs are most suitable and stable for HeApta modification and hemin addition. d) Calibration curve of Hemin-HeApta-SWCNT for different chelating agents. The isolated pyoverdines showed the lowest K_d = 0.26 µM (Binding fit, nonlinear curve fit (GraphPad Prism 8), data from Fig. 2f, (n = 3 independent experiments, mean ± SD).

Table ST1: Conditions for incorporation of SWCNTs into hydrogels (HG).

**Formula**	**Type-I**	**Type-II**	**Concentration**
PEG-DA (M_n = 700)	500 mg	500 mg	10 % (*m/V*)
PEG (6 kDa) (240 mg/ml in PBS)	-	2082 µL	10 % (*m/V*)
1xPBS	4296.6 µL	2215 µL	
UV-starter (2-Hydroxy-4′-(2-hydroxyethoxy)-2-methylpropiophenone, 12 mg/ml in H_2O)	208 µL	208 µL	0.5 mg/mL
SWCNT-dispersion in PBS	50 µL	50 µL	~ 0.3 absorption (6,5)-SWNCTs at ~ 990 nm

PBS and SWCNT-dispersion volumes were adjusted to obtain the desired nanosensor concentration. Egyptian blue -nanosheets (EB-NS) stock solution was diluted of 1:40 for the HG incorporation (125 µL EB-NS in water for 5 mL type-II HG).

a

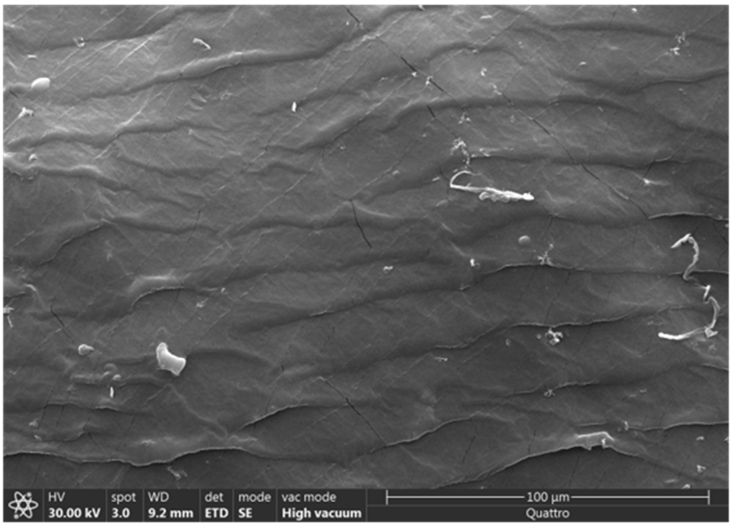

b

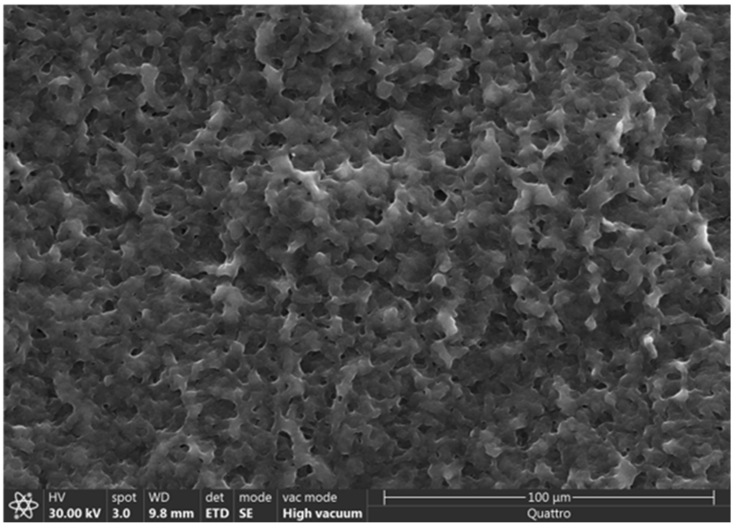

Figure S4: Scanning electron microscopy (SEM) of the sensor hydrogels.

a) Exemplary image of the type-I HG shows a uniform surface, while the porous type-II HG in b) displays a sponge-like structure. SEM was acquired with a LEO SUPRA 35 microscope (Zeiss) with an Inlens detector at 30 kV (secondary electrons). Hydrogel arrays were dialyzed in ddH_2O and lyophilized before gold sputtering (~4 nm layer). Experiments were repeated at least five times independently with similar results.

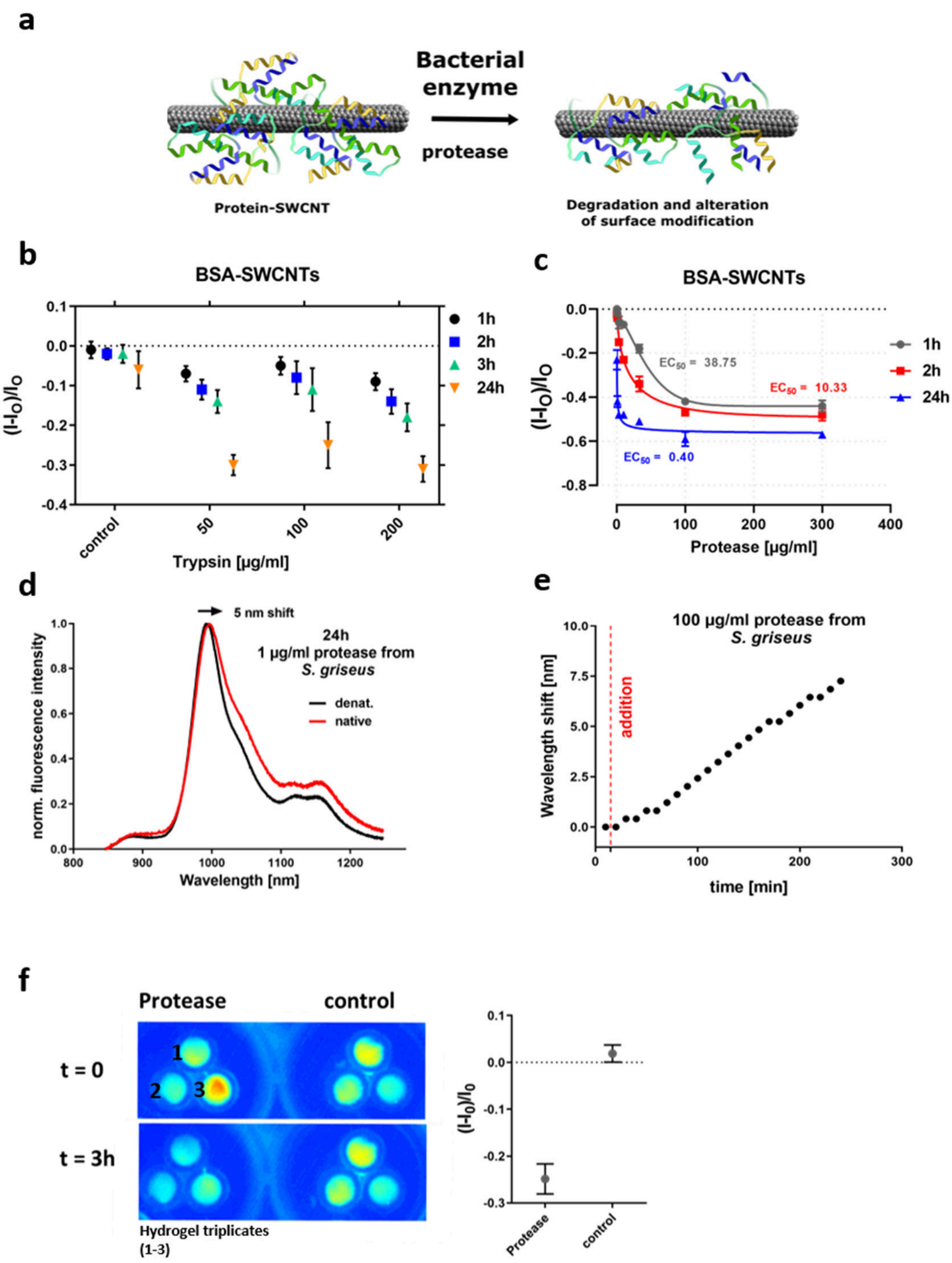

Figure S5: Extended characterization of BSA-SWCNTs as protease sensors.

a) Schematic of the protease sensing approach. The SWCNT surface is modified with a substrate (here the protein bovine serum albumin, BSA) for (bacterial) proteases, with the result that enzymes degrades and alters the organic phase around the SWCNTs, causing a fluorescence change of the nanosensor. b) BSA-SWCNTs in HG respond to trypsin (2.5% solution, 59427C, Sigma Aldrich) with a strong fluorescence decrease (control = 100 µg/mL thermally denatured trypsin in sodium dodecyl sulfate (SDS), n = 9 technical replicates in 3 independent experiments, mean ± SD). c) Calibration curves of BSA-SWCNTs in response to protease activity and corresponding EC_{50} (effective concentration) values (Dose-Response, nonlinear (asymmetric) curve fit (GraphPad Prism 8), data from Fig. 3b, (n = 9 technical replicates in 3 independent experiments, mean ± SD)). d) Fluorescence spectra of BSA-SWCNTs in HG after 24 h of incubation with 1 µg/mL protease from *Streptomyces griseus* show a 5 nm bathochromic shift for the native (active) enzyme (mean spectra of n = 3 independent experiments, see Figure 3 a,b). e) BSA-SWCNTs in HG after 3 h of incubation at room temperature with 100 µg/mL *Streptomyces griseus* protease: evaluation of the peak maxima from the (6,5)-SWCNTs emission around 1000 nm during enzyme performance. Dashed read line indicates the addition of protease. Within 3 h a peak shift of 7.5 nm was observed (n = 1). f) NIR stand-off images of the same experiment show a fluorescence decrease of ~25% (n = 3 independent experiments, mean ± SD) (control = PBS).

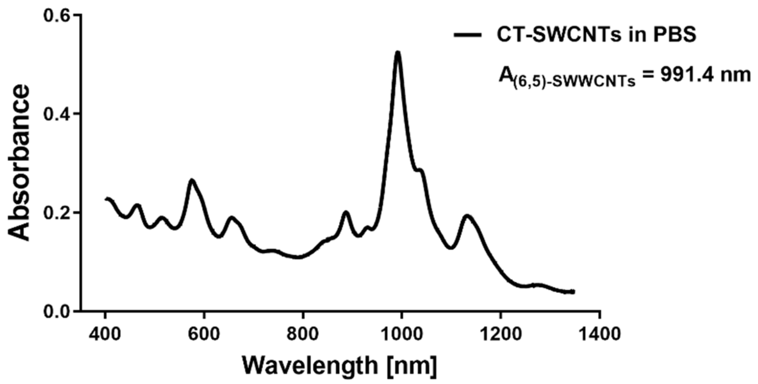

Figure S6: Absorbance spectra of CT-SWCNTs (nuclease sensor).

Calf thymus (CT)-SWCNTs 1:25 diluted in PBS. The E_{11} peak of the (6,5)-SWCNTs at 991.4 nm indicate well dispersed SWCNTs in denatured, long genomic CT-DNA.

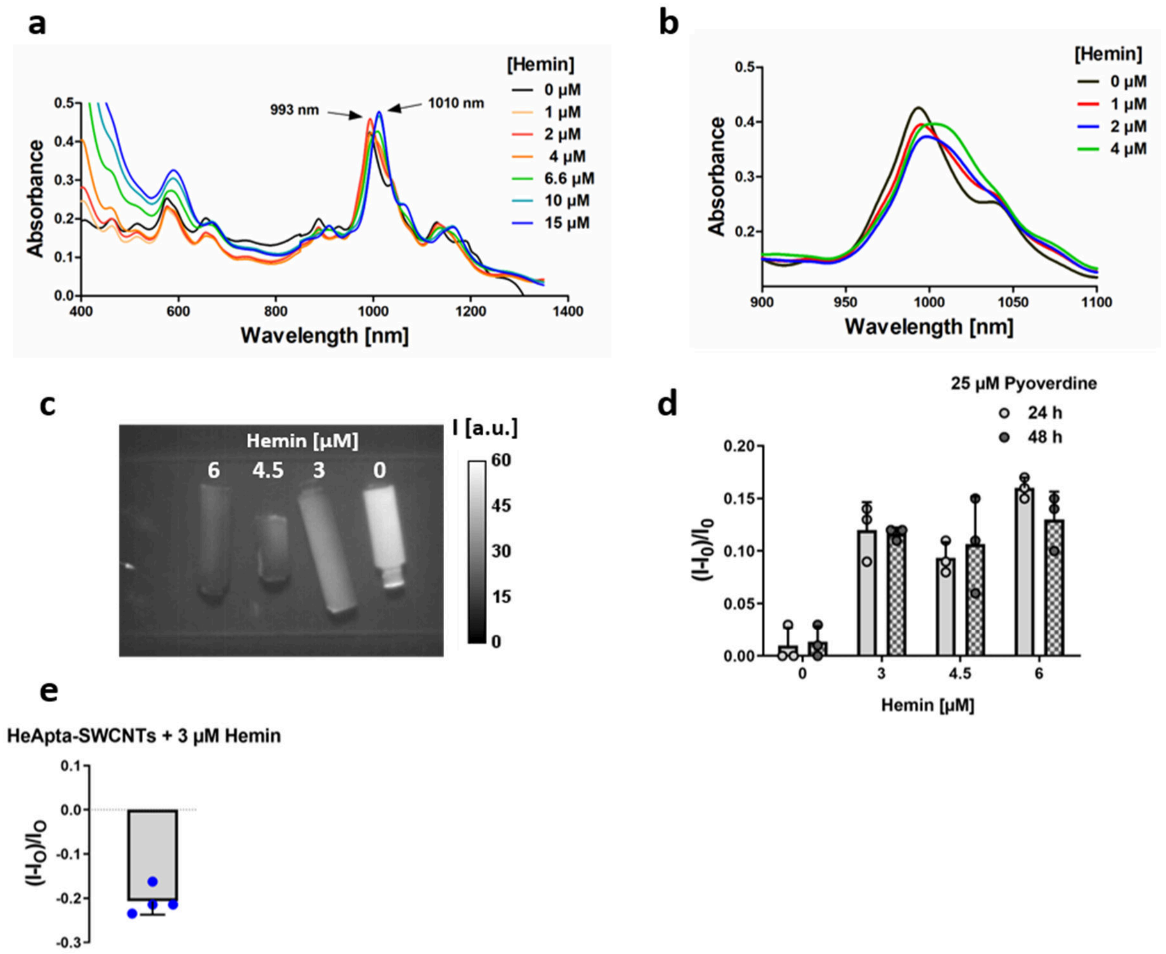

Figure S7: Integration of HeApta-SWCNTs in hydrogel.

a) Absorbance spectra of HeApta-SWCNTs in non-polymerized type-I HG. The addition of hemin redshifts the spectrum and results in visible aggregates that increase with hemin concentration (= loss of colloidal stability). b) Even for a hemin concentration ≤ 4 µM, redshift and broadening of the E_{11} transition of the HeApta-SWCNTs in the HG takes place and sensing of pyoverdine fails. Therefore, hemin addition before hydrogel polymerization was excluded. c) HeApta-SWCNTs in polymerized HG incubated with different hemin concentrations, to screen for optimal quenching and siderophore induced dequenching conditions. NIR stand-off image of HG-sensor cylinders show decreasing fluorescence intensities with increasing hemin concentration. The pyoverdine (25 µM) response in (d) is similar for all used hemin concentrations beforehand (n = 3 independent experiments, mean ± SD). Therefore, incubation in 3 µM hemin for 12 h, followed by HG dialysis in PBS for 2 days was used for all further HeApta-SWCNTs HG experiments. e) Fluorescence decrease of the HeApta-SWCNTs after incubation with the optimized hemin concentration (3 µM) (n = 4 independent experiments, mean ± SD).

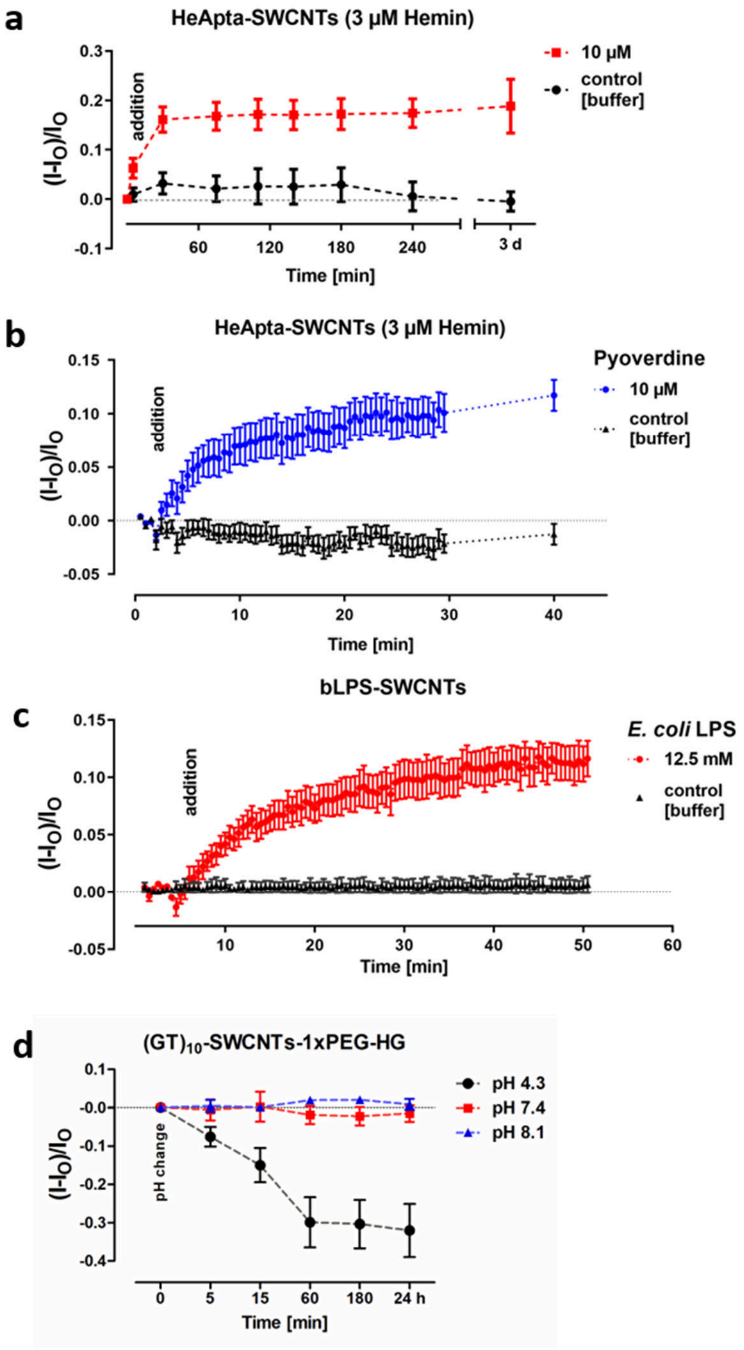

Figure S8: Time resolved fluorescence response of the sensor HGs to analytes.

a) The fluorescence readout of HG HeApta-SWCNTs (3 µM hemin) was evaluated *via* NIR stand-off detection. Addition of 10 µM pyoverdine (n = 3 independent experiments, mean ± SD) quickly increases the fluorescence and the response is not changing significantly for up to 3 days. b) A similar experiment with better time resolution shows that most of the response occurs in the first 10 min (n = 3 independent experiments, mean ± SD). c) Fluorescence response of bLPS-SWCNTs (n = 3 independent experiments, mean ± SD) to 12.5 µM *E. coli* LPS shows that most of the change happens in the first 20 min. d) Lowering the pH from 7.4 to pH 4.3, decreases the fluorescence of $(GT)_{10}$-SWCNTs HGs within 60 min by about -30%. pH increases to 8.1 do not significantly increase fluorescence compared to the control with pH 7.4 (n = 9 technical replicates in 3 independent experiments, mean ± SD).

Table ST2: Conditions for SWCNT surface modification

Nano-conjugate	Volume macromolecule	Volume PBS	Volume SWCNTs (2 mg/ml)	Tip sonication	Centrifugation	Removal of excess polymer
$(GT)_{10}$, C_{30} and $(GA)_{15}$ - SWCNTs	100 µL (2 mg/mL ssDNA in PBS)	100 µL	100 µL	15 min @ 36 W output power	2x 30 min @ 16100x *g*	Dialysis in HG
BSA-SWCNTs	100 µL (5 mg/mL BSA in PBS)	150 µL	50 µL	15 min @ 30 W output power	2x 20 min @ 16100x *g*	Dialysis in HG
HeApta-SWCNTs	100 µL (2 mg/mL aptamer in PBS)	100 µL	100 µL	15 min @ 36 W output power	2x 30 min @ 16100x *g*	Spin filtration
bLPS-SWCNTs	See protocol		75 µL	20 min @ 30 W output power	2x 30 min @ 16100x *g*	Spin filtration
CT-SWCNTs	100 µL (3 mg/mL ssDNA in PBS)	300 µL	50 µL	20 min @ 36 W output power	2x 30 min @ 16100x *g*	Dialysis in HG
PEG-PL-SWCNTs	See protocol		100 µL	20 min @ 36 W output power	2x 30 min @ 16100x *g*	Dialysis in HG

Dialysis in HG stands for the incubation (dialysis) of nanosensor HGs in PBS, removing non-reacted HG monomers, as well as excess polymers from SWCNT surface modification.

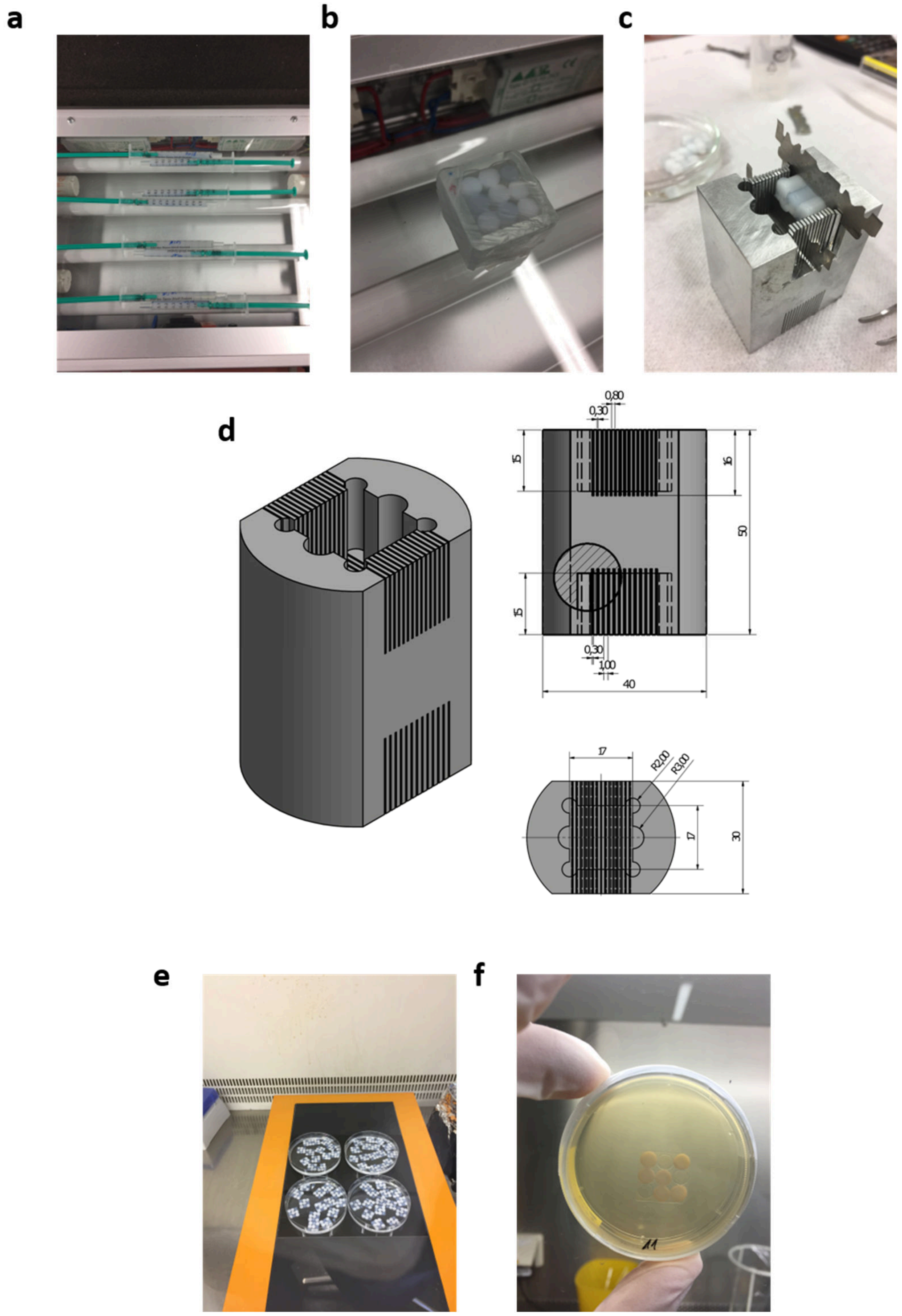

Figure S9: Engineering of the nanosensors hydrogel array.

a) Photograph of the hydrogel with nanosensors polymerized in a 1 ml syringe, which creates a uniform cylinder and excludes oxygen during the polymerization reaction. b) Assembly of different hydrogel sensor cylinders in a glass cube (1.5x 1.5x 1.5 cm, open at the two lateral planes). The cube is sealed with parafilm on one side, hydrogel cylinders are placed inside and the space in between filled with liquid type-I-HG (with 15% PEG-DA (*m/V*)). After complete sealing, the HG block is polymerized *via* UV illumination. c) Hydrogel array slices are generated by using a custom-made cutting chamber with razor blades. This way, uniform 0.8 mm thick slices of sensor array hydrogels are created. d) Detailed information on the alumina HG cutting chamber. e) UV sterilization of the HG array slides guarantees contamination free conditions for microbiological experiments. f) Sterilized HG array embedded in microbial agar.

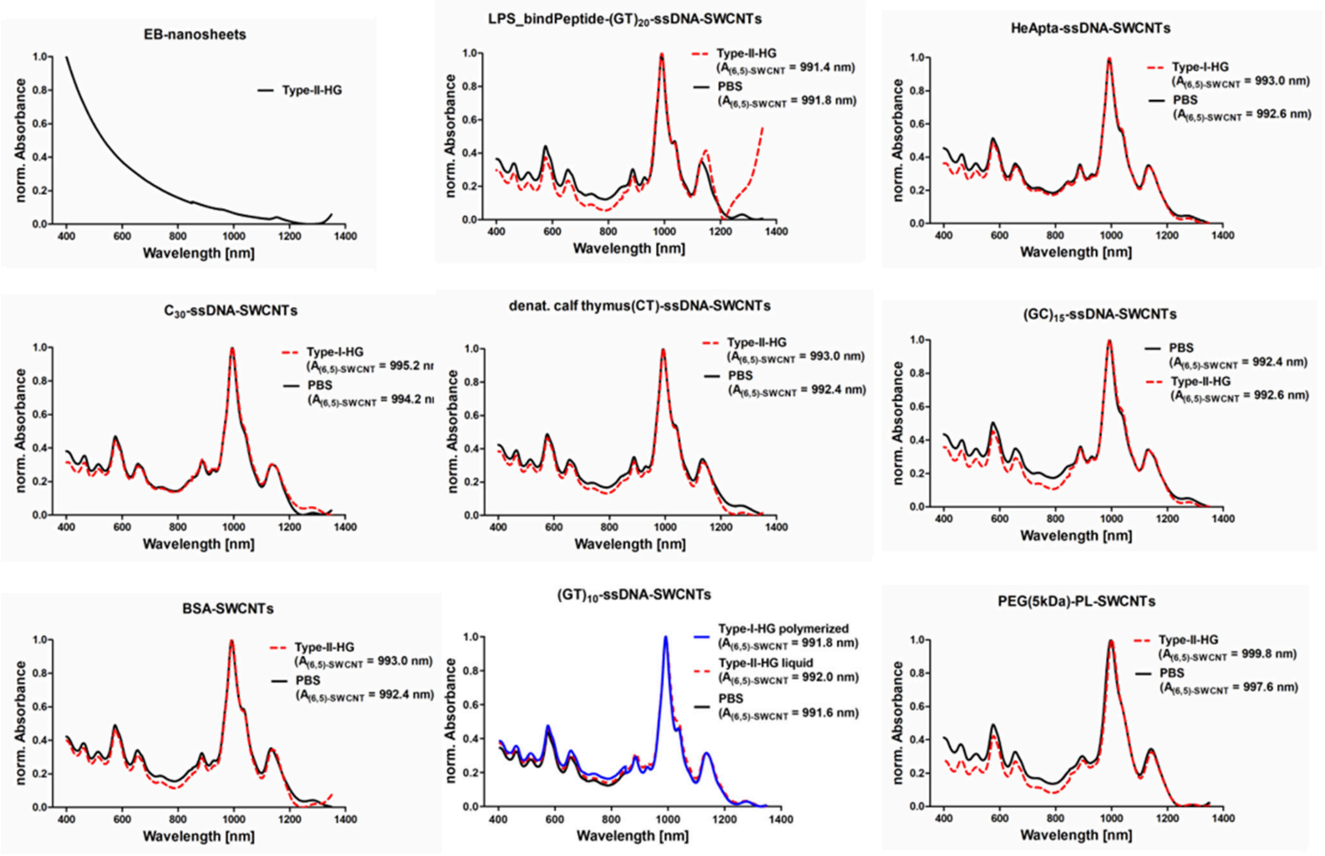

Figure S10: Absorbance spectra of the sensor array components in solution and in hydrogel.

Normalized absorbance spectra of the nanosensors of the sensor array, before and after incorporation in the HG. No major shifts were noticed, indicating colloidal nanosensor stability even in the hydrogel environment.

Table ST3: Overview nanosensor array

Acronym	Hydrogel	Nano-conjugate	Main target	Reference
EB-NS	Type-II	Egyptian blue nanosheets	nIR reference	[2]
bLPS	Type-II	$(GT)_{20}$-ssDNA-linker-LPS-binding peptide	Lipopolysaccharides (LPS)	This study
HeApta	Type-I	Hemin-aptamer-SWCNTs (+hemin)	Iron chelating siderophores	This study
C_{30}	Type-I	C_{30}-ssDNA-SWCNTs	Unspecific, pH	[3]
CT	Type-II	Genomic, denatured calf thymus (CT) ssDNA-SWCNTs	Nucleases	This study
$(GC)_{15}$	Type-II	$(GC)_{15}$-ssDNA-SWCNTs	Unspecific, pH	[3]
BSA	Type-II	Protein modified BSA-SWCNTs	Protease	This study
$(GT)_{10}$	Type-I	$(GT)_{10}$-ssDNA-SWCNTs	pH, O_2 concentration	[3,4]
PEG	Type-II	Phospholipid (18:0 PEG5000PE) DSPE-PEG(5000) -SWCNTs	Proteins	[5,6]

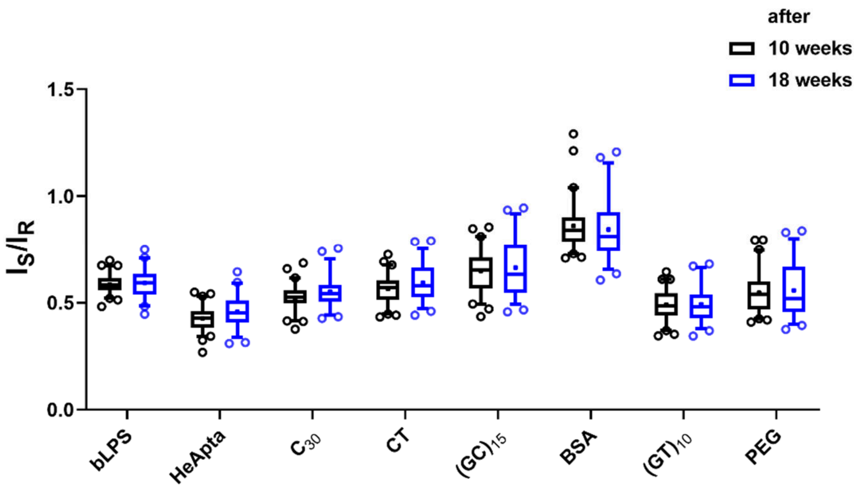

Figure S11: Stability of the nanosensor hydrogel array.

The intensity ratios for HG embedded nanosensors are shown as Box-plots (5 - 95% percentile). The fluorescence intensities of the sensor spots (I_S) are shown relative to the fluorescence intensity of the NIR reference material (EB-NS) (I_R). The ratios of in total 60 arrays (n = 60 independent experiments), evaluated after 10 weeks of nanosensor-HG manufacturing and storage in PBS, are shown in black and do not differ significantly from the ratios of in total 55 arrays (n = 55 independent experiments) shown in blue, assembled 2 months later from the same nanosensor HGs (25-75 percentile box with median as bar and mean as square, 5-95 percentile whiskers, individual datapoints outside the 5-95 percentile as dots). This result indicates a high stability of the nanosensors in the HG matrix over several months, as degradation or loss of colloidal stability would change the fluorescence emission of the nanosensors relative to the reference. No significant differences between HG type or SWCNT surface modification (ssDNA, proteins or PEG-phospholipid) was observed.

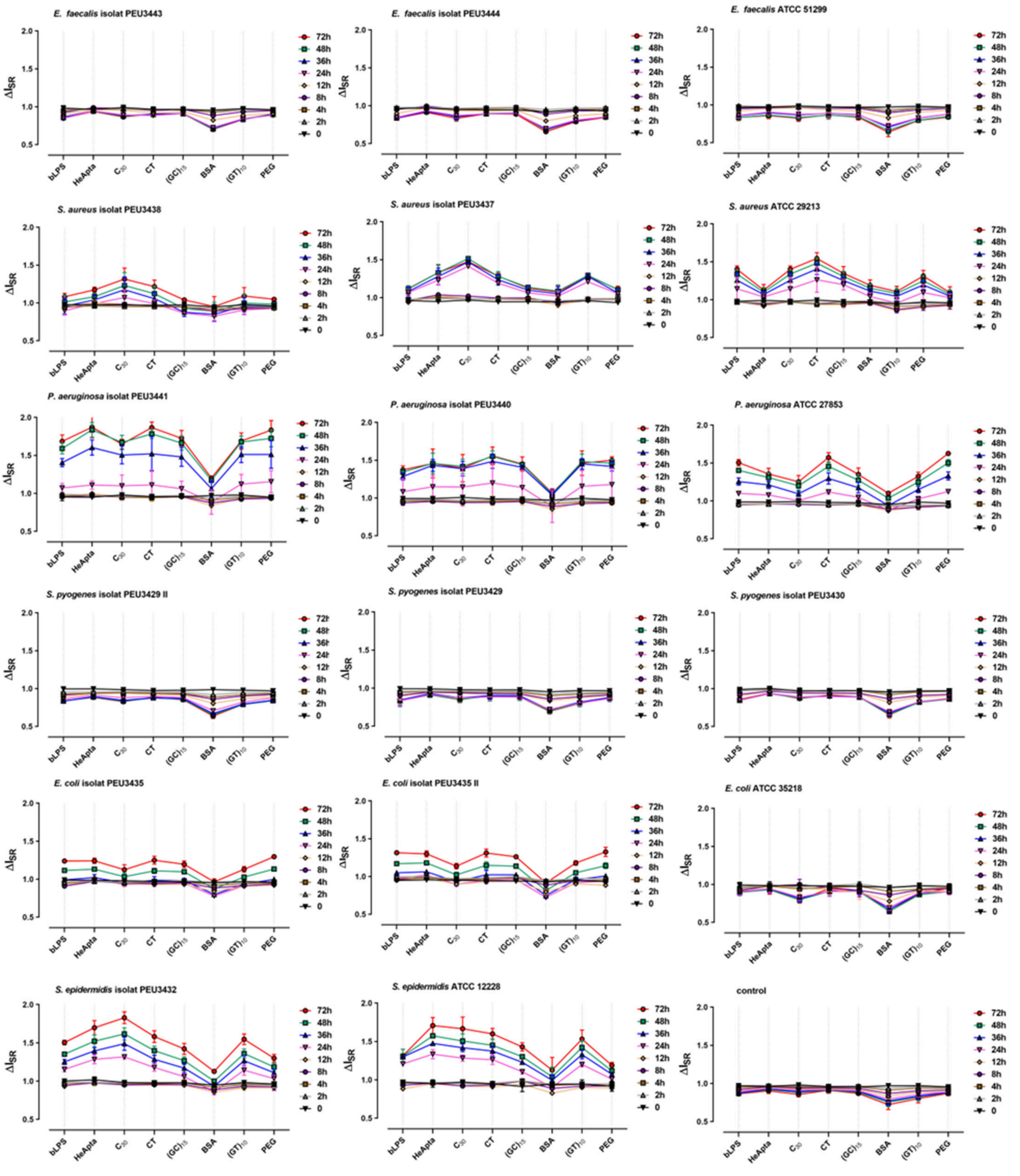

Figure S12: Bacterial sensor array fingerprints.

Corresponding to Fig. 4d-h, time resolved bacterial fingerprints are presented for different bacteria. The different sensor arrays responses indicate major differences between some of the tested pathogens (n = 9 technical replicates in 3 independent experiments, mean ± SD).

a

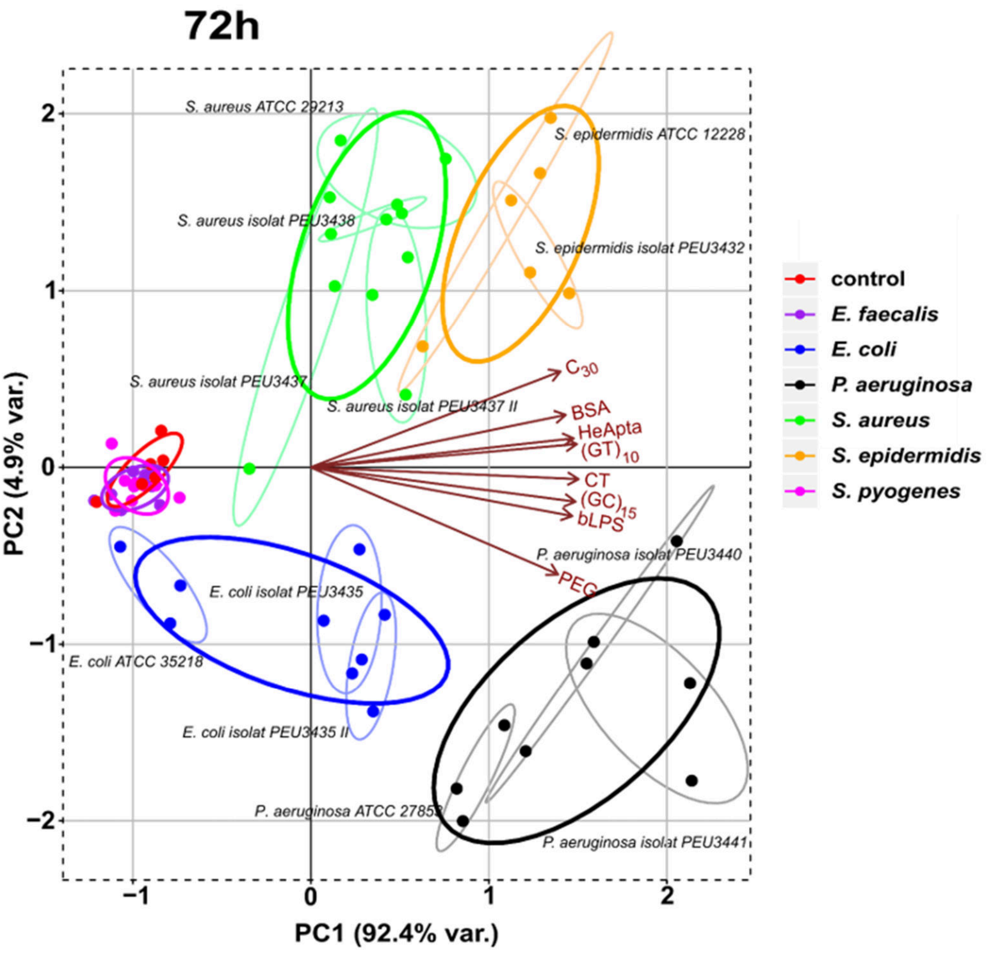

b

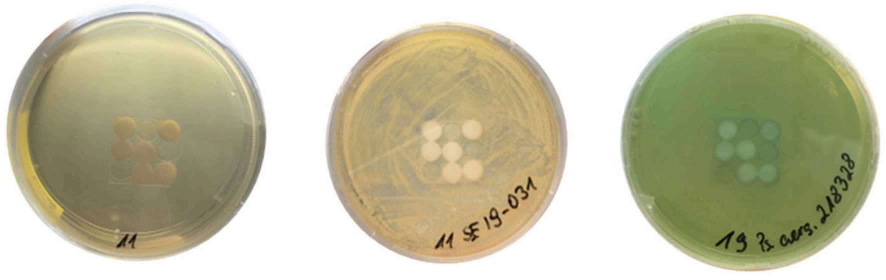

Figure S13: Principal component analysis (PCA) based differentiation of bacteria with indicated strains.

a) Sensor array fingerprints lead to the PCA differentiation clusters shown in Fig. 4h, but the different isolates of the same species were not shown, for reasons of clarity and comprehensibility. Here, a more detailed representation for the timepoint 72 h after incubation is presented. Next to the different species, also strain/isolates sub-clusters, as well as the variables (as vectors) for the different sensor responses are indicated. The spread of the datapoints within the cluster of one bacterium is therefore mainly attributed to the epigenetically different strains and not due to a large spread within the sub strains (n = 3 technical replicates). b) Photographs of the sensor arrays incorporated in agar: on the left side, before bacterial inoculation (equal to control without bacterial growth), in the middle of *S. epidermidis* and on the right of *P. aeruginosa (isolate PEU3440= specimen 218328)* after 72 h.

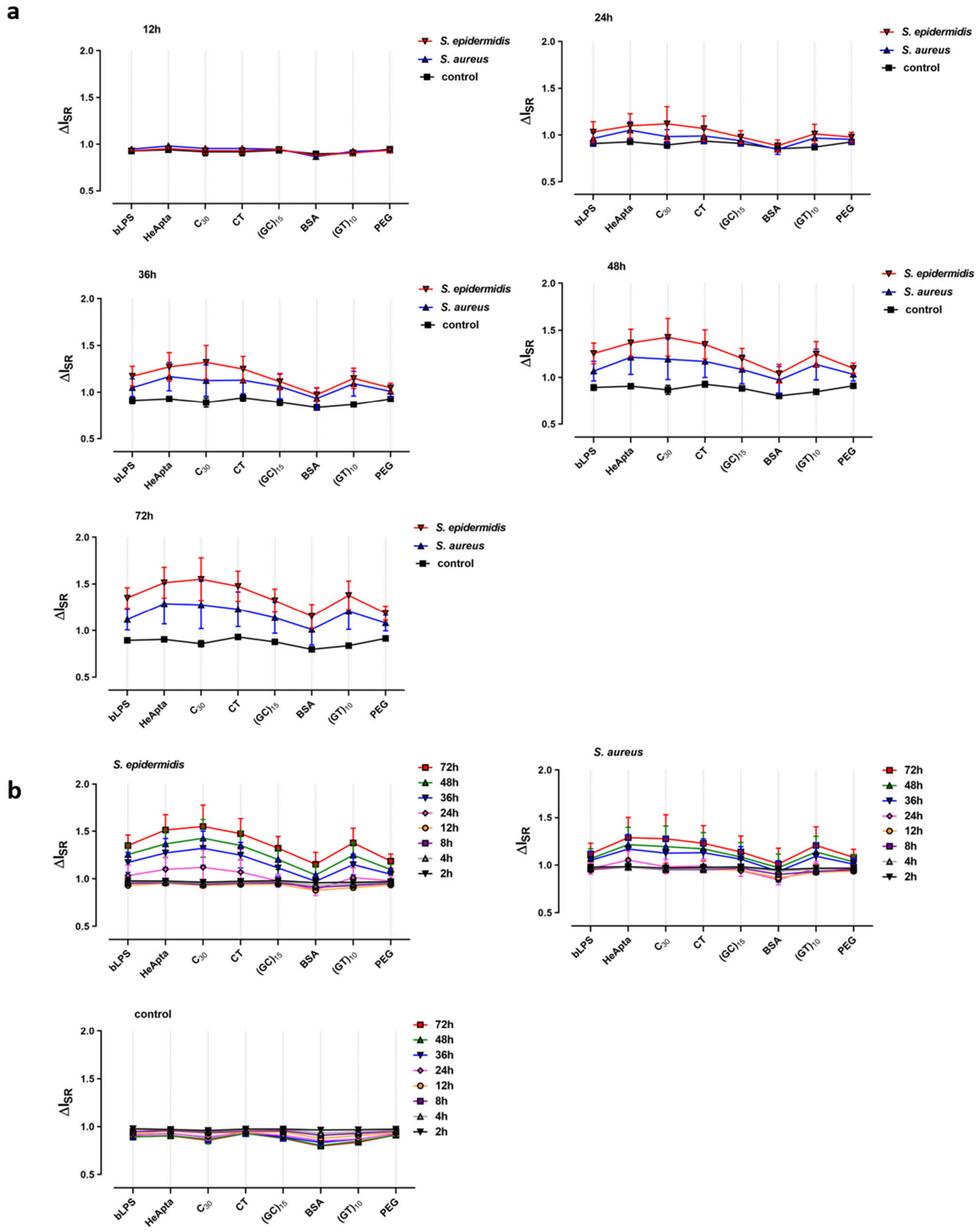

Figure S14: Bacterial fingerprints from *S. aureus* and *S. epidermidis*.

Corresponding to Figure 4i, time resolved bacterial fingerprints are presented. a) Comparison between the mean sensor readout from clinical isolates from *S. aureus* (n = 21 biologically independent samples) and *S epidermidis* (n = 22 biologically independent samples) at different timepoints after inoculation (control n = 3 independent experiments) (mean ± SD). b) Overview about the sensor fingerprint evolution during the experiment. Note that the 'error' reflects the spread within the clinical isolates and not a technical error (same samples as presented in a).

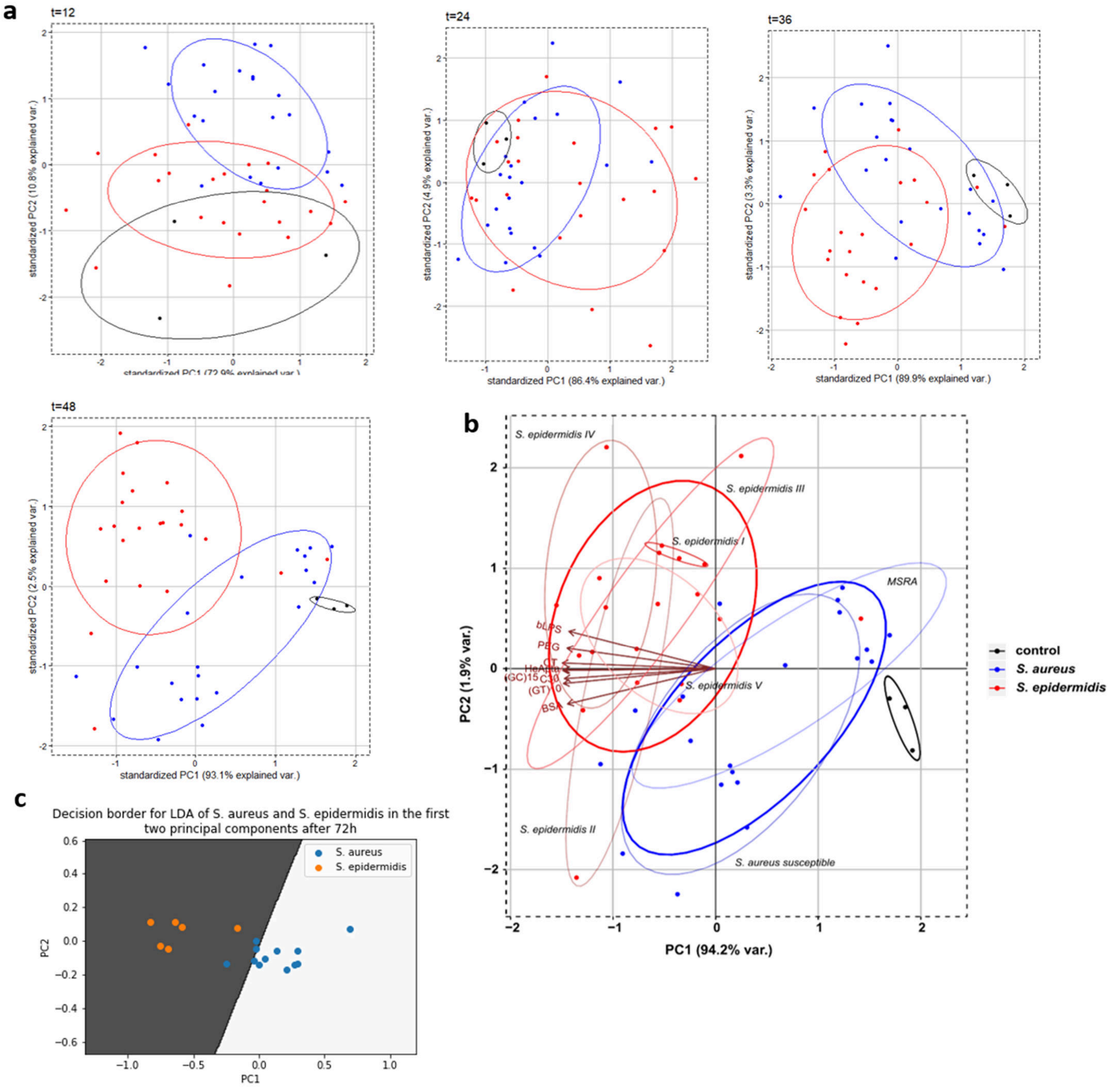

Figure S15: PCA for sensor fingerprint of *S. aureus* and *S. epidermidis*.

a) PCA of the array fingerprints of clinical isolates of *S. aureus* and *S epidermidis* (43 independent clinical isolates) at different timepoints. The two clusters separate after 48 -72 h. b) Sub-clusters of isolates are indicated for *S. epidermidis*, and *S. aureus* including antibiotic susceptibility, as well as the variables (as vectors) for the different sensor responses, 72 h after incubation. c) Linear discriminant analysis for the classification of *S. aureus* and *S. epidermidis* from Figure S12 by training data set based on the 43 clinical isolates shown above. The result indicates, that independent isolates can be distinguished, classified and assigned to its bacterial group with a > 80 % likelihood.

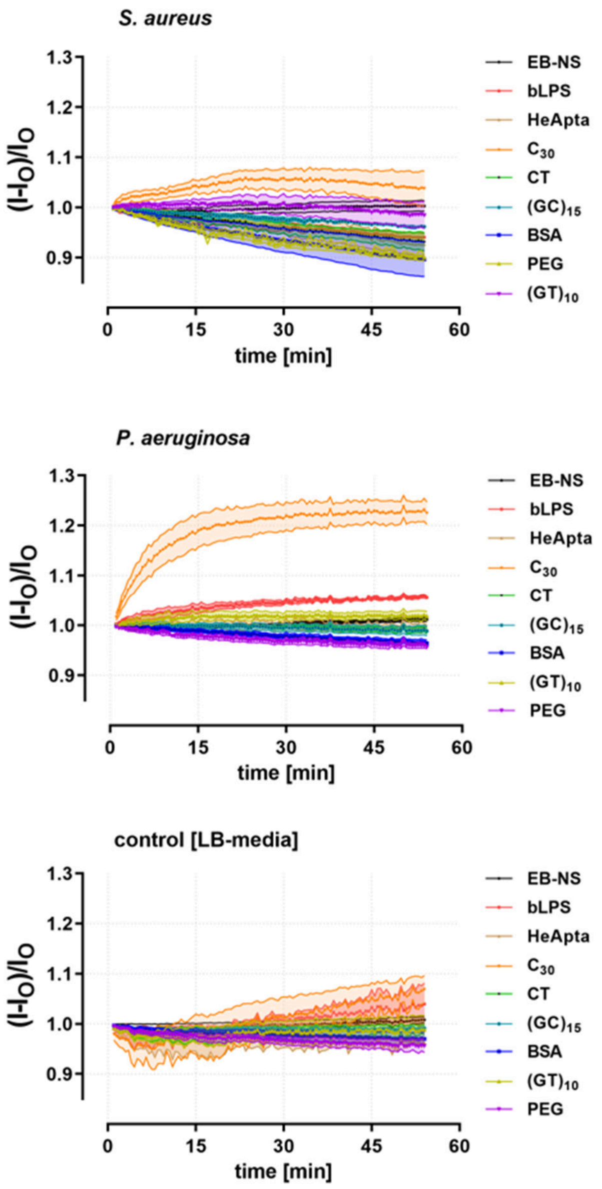

Figure S16: Response kinetics of sensor array.

Time resolved mean fluorescence change, with SD (pale boundaries), of the nanosensors in the hydrogel array after addition of liquid culture supernatant from *S. aureus*, *P. aeruginosa* and LB-media as control. (24 h incubation in LB-medium, I- intensity sensor at timepoint t, I_0- intensity sensor at t = 0) (n = 3 independent experiments, mean ± SD). Note: For reasons of clarity, the mean time resolved changes are shown in Fig. 4k. Comparison with LB-media response shows a clear difference of the sensor array response.

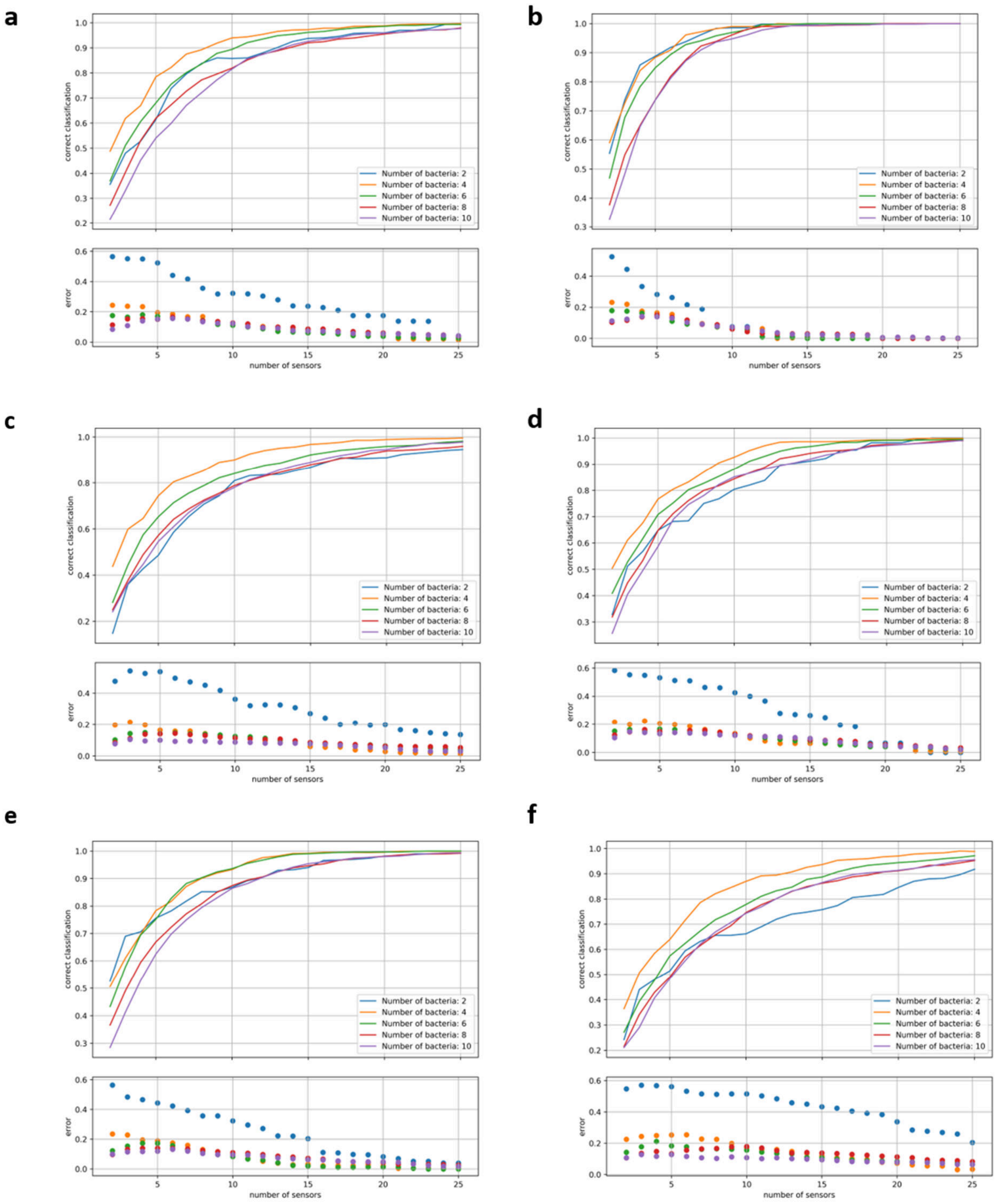

Figure S17: Sensor array response simulation.

a) Simulation with the parameters, extracted from the original HG sensor experiments (noise = 10 %, indistinguishable sensor responses = 40 %, sensor responses = 0.7 – 1.7) (note = same data as Figure 5f, shown here again for reasons of comparability). b) Increasing the sensor response to 0.5 – 2 would strongly enhance the bacterial differentiation, while other parameters stay fixed (noise = 10 %, indistinguishable sensor responses = 40 %, sensor responses = 0.5 – 2). c) noise = 5 %. d) noise = 15 %. Changing the noise level dos not significantly change the simulation output (indistinguishable sensor responses = 40 %, sensor responses = 0.7 – 1.7). e) Simulation with less indistinguishable sensor responses lead to a decreased number of necessary sensors for bacteria differentiation (noise = 10 %, indistinguishable sensor responses = 20 %, sensor responses = 0.7 – 1.7). f) Simulation with more indistinguishable sensor responses lead to an increased number of necessary sensors for bacteria differentiation (noise = 10 %, indistinguishable sensor responses = 60 %, sensor responses = 0.7 – 1.7). The results show that both, more specific sensor responses and an increased number of sensors improves bacteria differentiation and identification (e.g. for more pathogen species or further evaluation on the subspecies level).

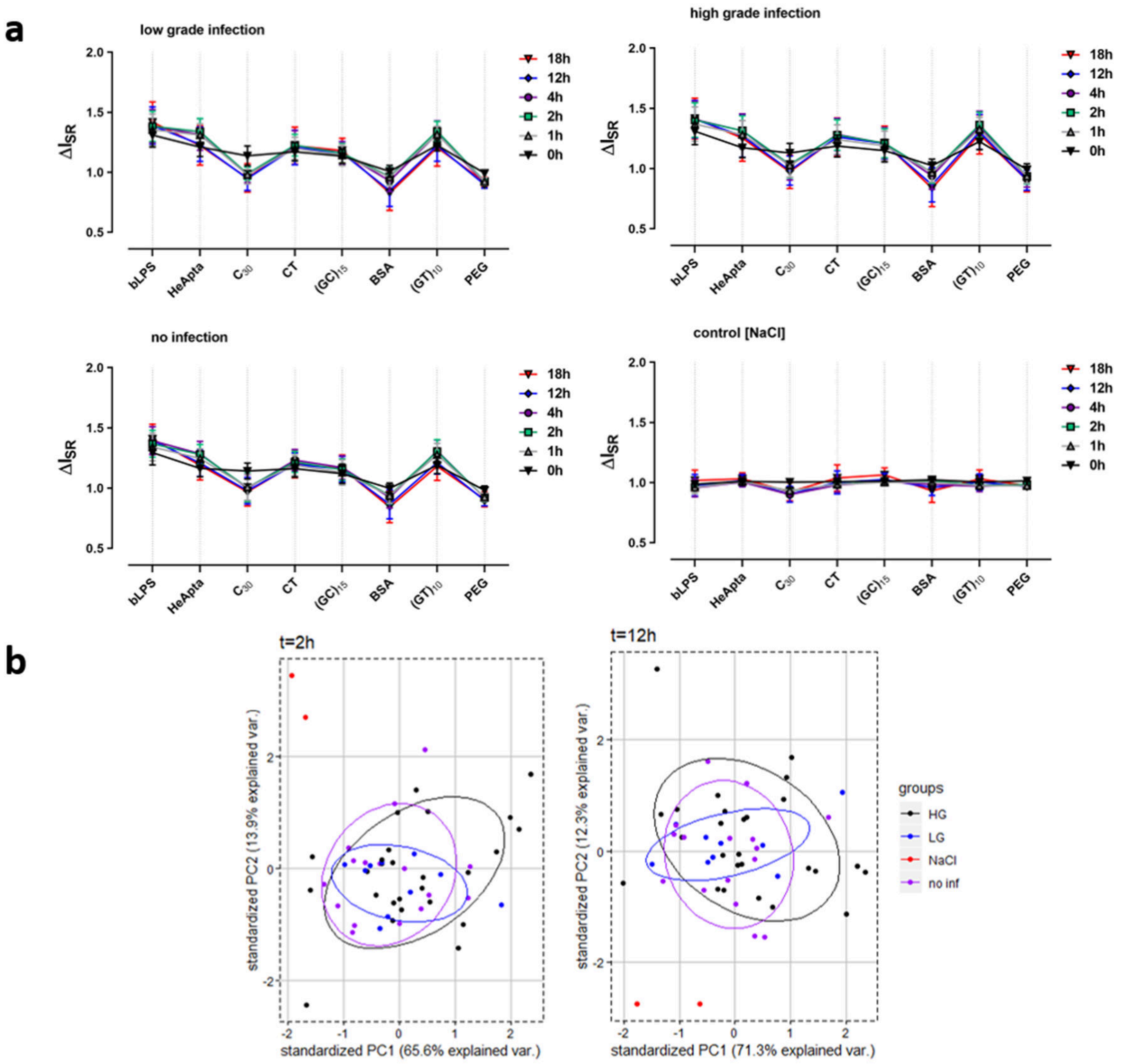

Figure S18: Human joint fluid (synovia) influence on sensor response.

Sensor array responses to human joint fluids from different patients (n = 26 biologically independent samples). Fluid from non-infected patients and low/high-grade infections were applied to the sensor array to evaluate the impact of different immune responses and synovia compositions. a) Sensor array fingerprints for synovia fluid samples for different timepoints show uniform pattern, indicating that synovia from diverse human background does not unspecifically bias the sensor array response (mean ± SD, for n see table ST4). b) Corresponding PCs do not show differences for various synovia types and samples from diverse patient backgrounds. These results indicate robustness of the sensor array performance towards a future smart implant application.

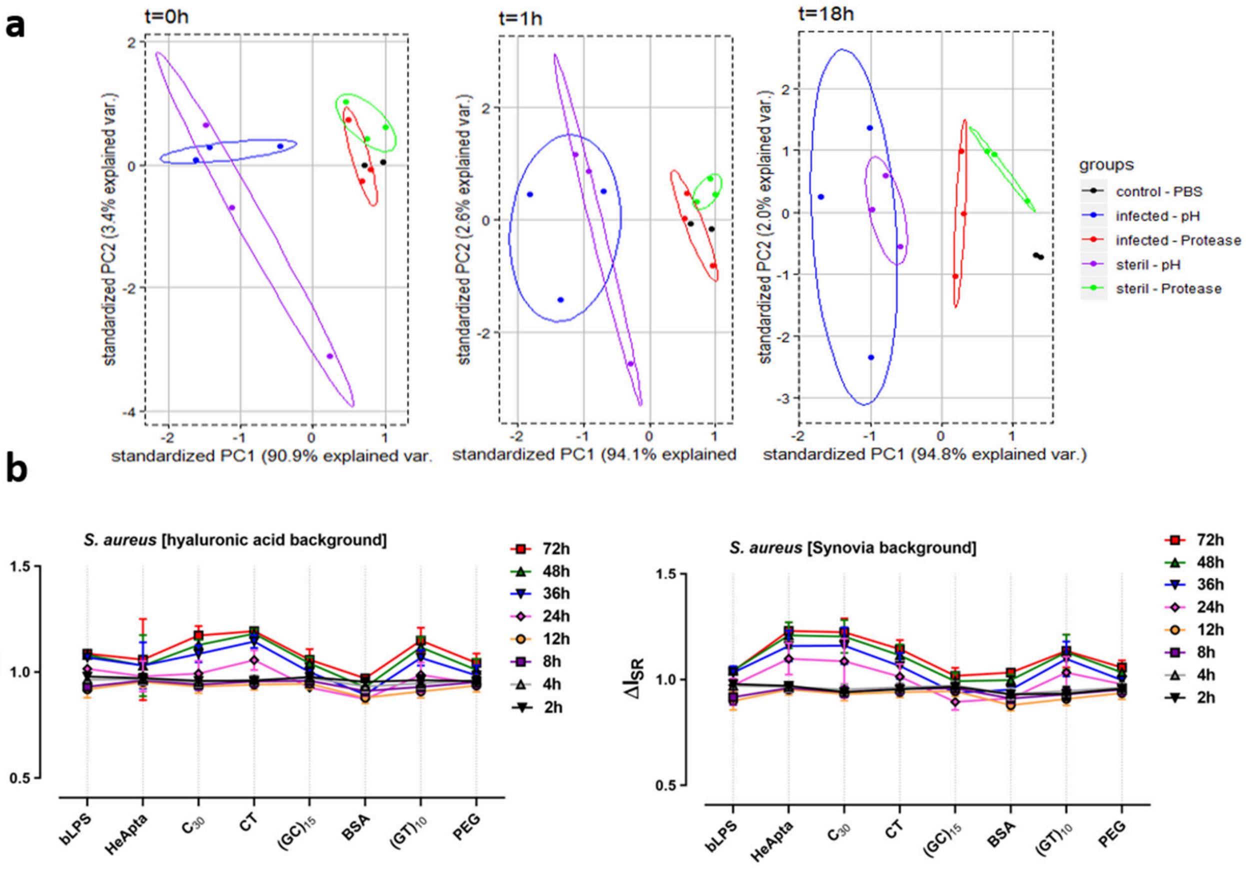

Figure S19: Sensor array performance in synovia background.

a) Sensing of pH changes and protease activity in human knee fluid background. Sensor arrays show a direct response to change of pH to 4.5. Protease activity requires longer to be detected. Differences between infected and non-infected knee-fluid are apparent, probably due to different amounts of proteins present *via* prevalent immune response. b) Sensing of methicillin resistant *S. aureus* (MRSA) during bacterial growth, in a microbiological agar after incubation / with background of hyaluronic acid (main component of the synovia) and the synovia itself from non-infected patients (n = 3 independent experiments, mean ± SD). The array is able to sense bacterial targets and bacterial growth, even in complex matrixes such as synovia. Therefore, only direct biofilm infections on a potential smart implant surface would be sensed by the nanosensor array.

Table ST4: Human joint fluids.

Number	Infection state	Pathogen	pH synovia
1	HG	Coagulase negative Staphylococci	7.5
2	HG	*Staphylococcus epidermidis*	7.5
3	HG	*Staphylococcus epidermidis*	7.5
4	HG	*Candida albicans*	7.5
5	HG	*Staphylococcus lugudensis*	7.0
6	HG	*Streptococcus agalactiae*	8.0
7	HG	Not detected	8.0
8	HG	*Staphylococcus aureus*	7.0
9	HG	Not detected	8.0
10	HG	Lactobacillus	7.0
11	HG	*Escherichia coli*	8.0
12	HG	*Staphylococcus aureus*	6.5
13	LG	Not detected	7.0
14	LG	Not detected	8.0
15	LG	Not detected	7.0
16	LG	Not detected	7.5
17	LG	Not detected	7.0
18	Sterile	Sterile	7.5
19	Sterile	Sterile	7.5
20	Sterile	Sterile	7.5
21	Sterile	Sterile	7.5
22	Sterile	Sterile	7.0
23	Sterile	Sterile	7.0
24	Sterile	Sterile	7.0
25	Sterile	Sterile	7.0
26	HG	Propiobacterium	7.5

HG- high grade, LG- low grade infection

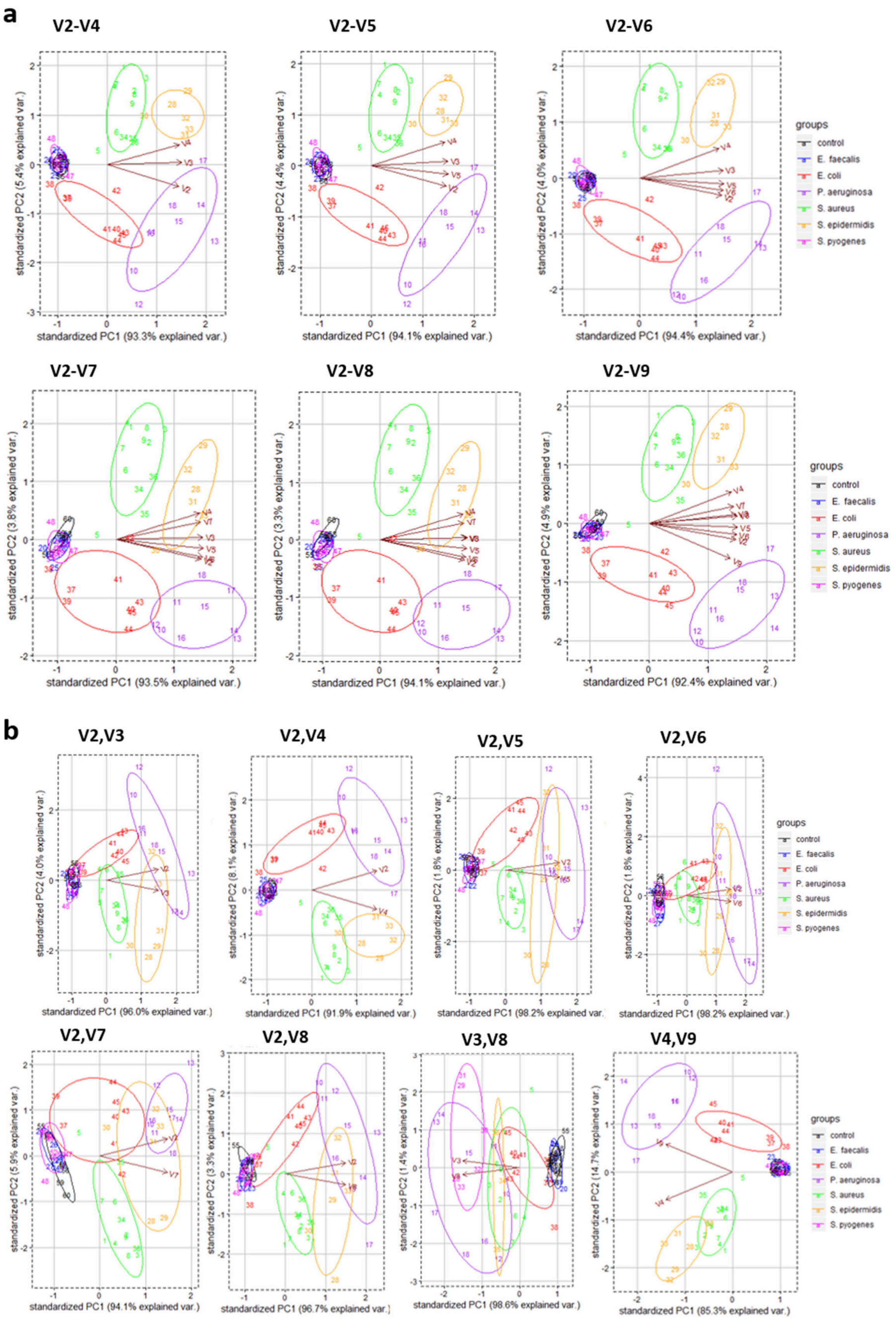

Figure S20: PCA based pathogen differentiation with reduced number of sensors.

All graphs are based on the sensor fingerprint after 72 h (Fig. 4h). Numbers indicate single biological samples. Vectors display the influence of the variables (V) as different sensors responses; V2: bLPS-SWCNTs, V3: HeApta-SWCNTs, V4: C_{30}-SWCNTs, V5: CT-SWCNTs, V6: $(GC)_{15}$-SWCNTs, V7: BSA-SWCNTs, V8: $(GT)_{10}$-SWCNTs and V9: PEG-SWCNTs; all related to EB-NS. a) Comparison of using different sensor responses for the PCA from V2 to V9 shows the principle bacteria differentiation is even possible with 3 sensors (V2-V4). b) Different combinations for V2 and another sensor response. Herby, the combination with V4 (C30) and V9 (PEG) showed a promising differentiation of the bacteria, while using the response of two sensors. The best separation was found for V4 with V9. V3 and V8 is exemplarily shown for all sensor combinations, which does not lead to cluster separation.

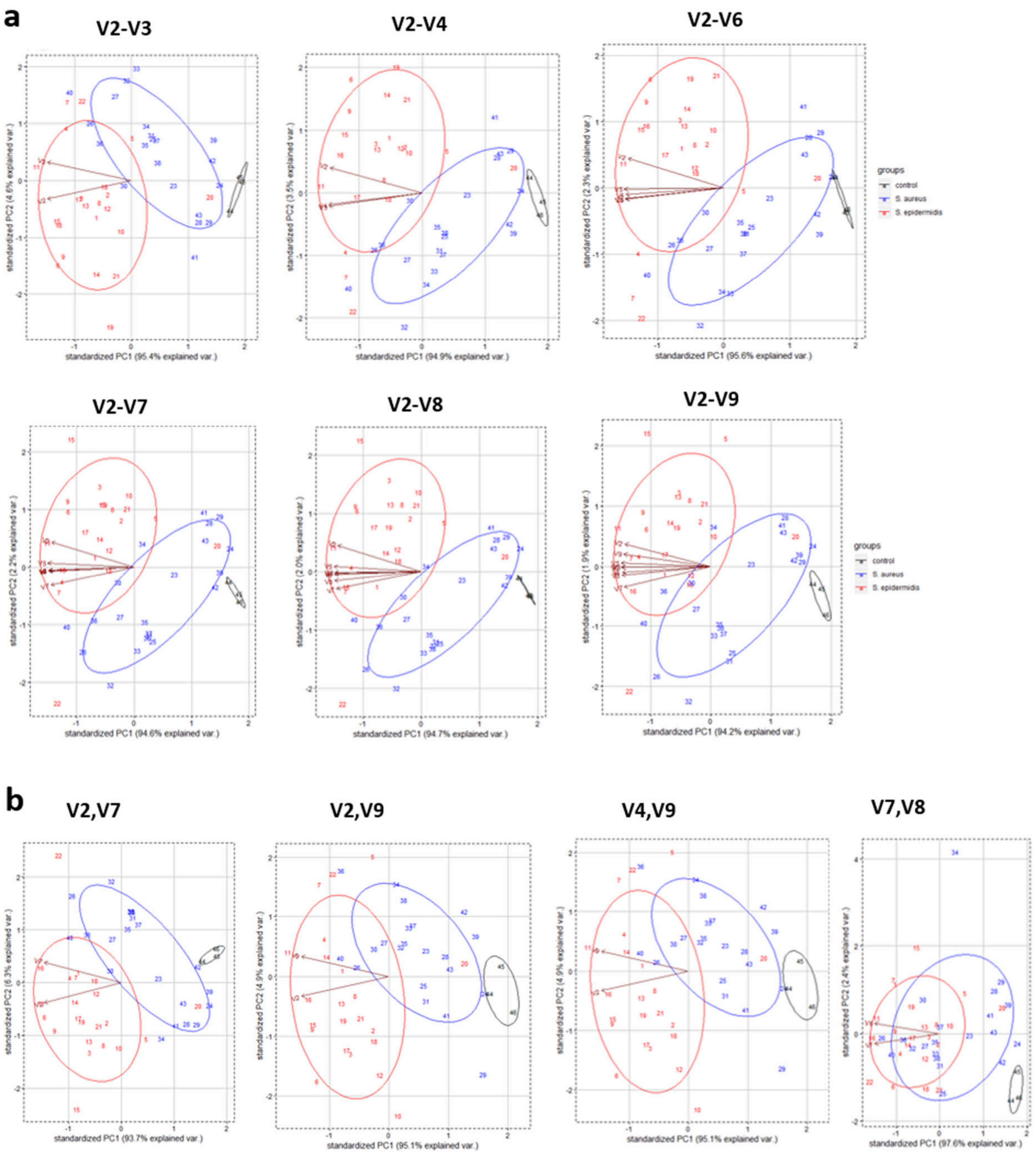

Figure S21: PCA differentiation of *S. aureus* and *S. epidermidis* with reduced number of sensors.

All graphs are based on the sensor fingerprint after 72 h (Fig. 4j). Numbers indicate single biological samples. Vectors display the influence of the variables (V) as different sensors responses; V2: bLPS-SWCNTs, V3: HeApta-SWCNTs, V4: C_{30}-SWCNTs, V5: CT-SWCNTs, V6: $(GC)_{15}$-SWCNTs, V7: BSA-SWCNTs, V8: $(GT)_{10}$-SWCNTs and V9: PEG-SWCNTs; all related to EB-NS. a) Comparison of using different sensor responses for PCA from V2 to V9 shows that differentiation of both strains is possible with two to three sensors (V2-V4). b) Different combinations of two sensor responses indicating for V2 and V7 or V2 and V9 a strong separation, while V4 and V9 or V7 and V8 generating a large overlap of the two bacteria, within the 43 tested isolates.

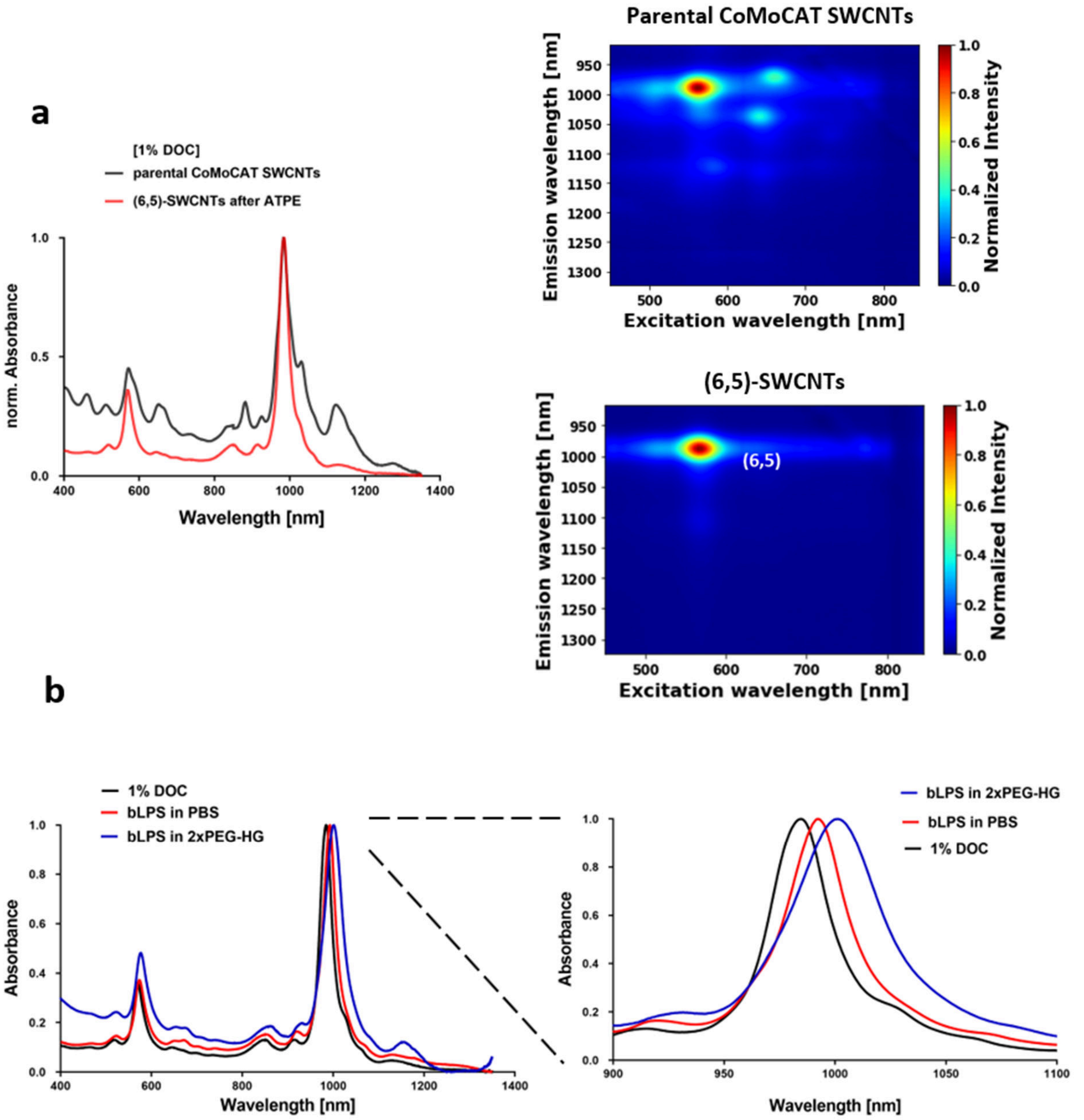

Figure S22: (6,5)-SWCNT purification and surface exchange for LPS sensing.

a) Absorbance spectra of (6,5)-SWCNTs and its parental CoMoCAT SWCNTs. Aqueous two-phase separation (ATPE) yields quasi monochiral (6,5)-SWCNT by following the protocol from Li *et al.*[7]. 2D excitation emission fluorescence spectra of the purified (6,5)-SWCNTs show the clear absence of other fluorescent SWCNT chiralities, compared to their parental SWCNT solution. b) These purified (6,5)-SWCNTs in 1% DOC were exchanged[8] to the LPS-bind-peptide-$(GT)_{20}$ (bLPS), which causes an absorbance shift from 985 nm to 992 nm. Incorporation into type-II-HG redshifts the absorption peak to 1001 nm.

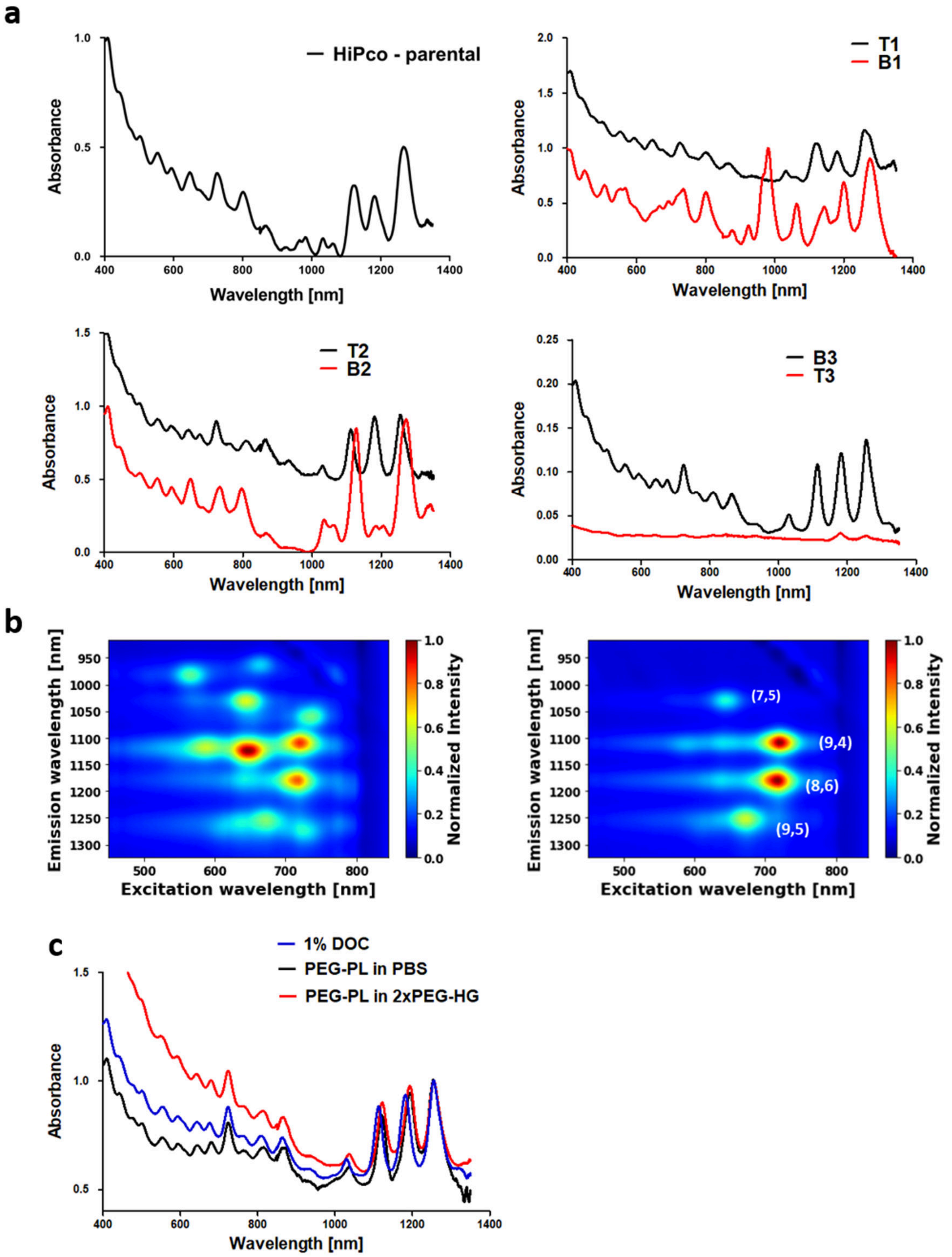

Figure S23: ATPE purification from HiPco-SWCNTs and surface exchange to PEG-PL surface modification.

a) UV-Vis-NIR absorbance spectra for the several ATPE steps. The goal was to obtain a SWCNT sample, which lacks the small diameter fluorescent SWCNTs to avoid overlap with (6,5)-SWCNTs. Most importantly, in the first separation step, the bottom phase (B1) gets enriched with SWCNTs, absorbing below 1100 nm. In the second step, another fraction of undesired, smaller diameters gets excluded, while in the third step, the purified SWCNTs are transferred and therefore concentrated to the bottom (B3) phase. b) The 2D excitation emission fluorescence spectra show three major, large SWCNT chiralities (9,4), (8,6) and (9,5), next to a smaller fraction of (7,5)-SWCNTs, compared to the parental solution, which contains multiple, also smaller diameter SWCNTs. c) Surface exchange from 1% DOC to DSPE-PEG(5000) (PEG-PL) leads to a shift in absorbance for all SWCNT chiralities, except (9,5)-SWCNTs. This separation provides access to large-diameter SWCNTs with a fluorescence emission >1100 nm.

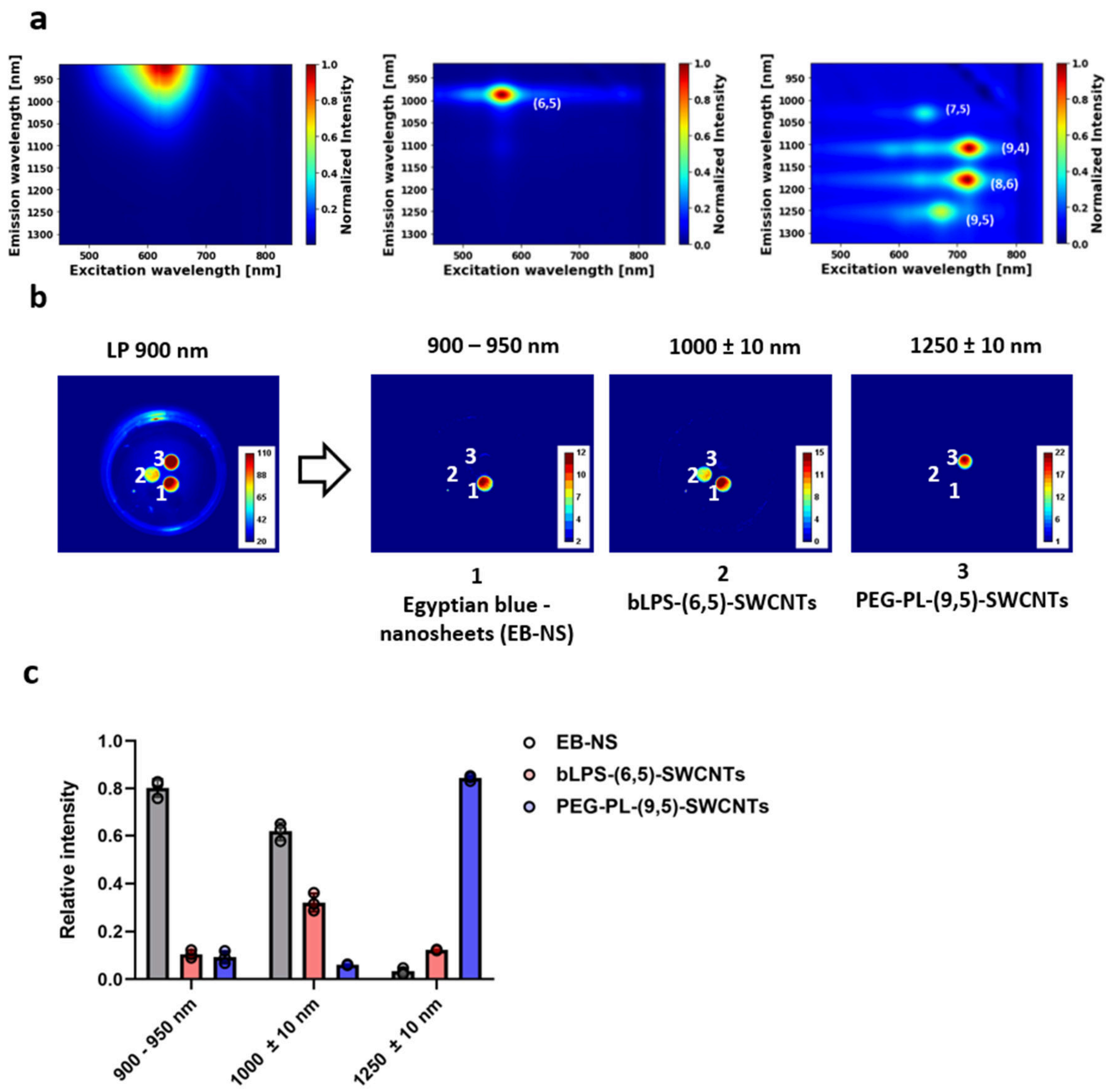

Figure S24: Characterization of sensors for hyperspectral sensing.

a) 2D spectra of the used NIR fluorophores: EB-NS, (6,5)-SWCNTs and large diameter HiPco-SWCNTs. b) NIR fluorescence stand-off image of three different HG sensors (left- 900 nm LP filter). Different used filters allow differentiation of the spectral encoded sensors, each one in a single type-II HG. c) Evaluated spectral overlap for the different HG sensors, separated by the used emission filters. EB-NS and PEG-PL-SWCNTs show a very small overlap, while the bLPS-(6,5)-SWCNTs sensor signal contains a (constant) EB-NS background (n = 3 independent experiments, mean ± SD).

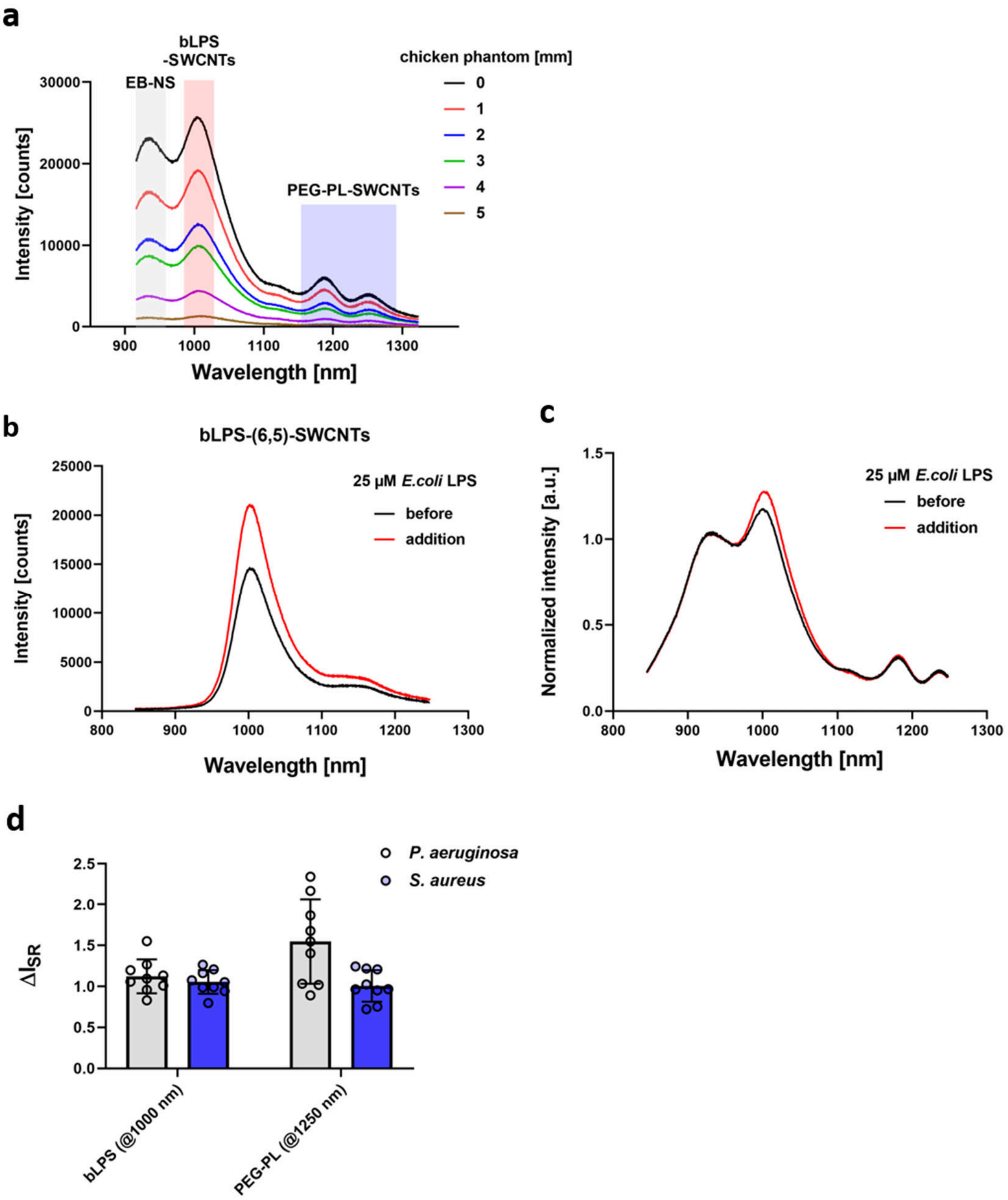

Figure S25: Hyperspectral sensing.

a) Decrease of fluorescence emission for increasing chicken phantom tissue thickness. b) Monochiral bLPS-(6,5)-SWCNT sensor reaction response to LPS addition with a fluorescence increase, which is less prominent if compared to the one from non-purified bLPS-sensors in Fig. 2. c) LPS addition to hyperspectrally encoded sensor HG leads to a small increase. d) Sensor response of the hyperspectral encoded sensors, referenced to EB-NS (900-950 nm) intensity, after 72 h of incubation with *P. aeruginosa* and *S. aureus* (n = 9 independent experiments with 3 biologically independent samples, mean ± SD) (see Fig. 5c).

Table ST5: Bacteria strains I.

Species	Designation	Gender	Age	Year	Source material
S. aureus	ATCC 29213	-	-	-	-
S. aureus	PEU3437	male	66	2019	other
S. aureus	PEU3438	male	7 mts	2019	nasal swab
Ps. aeruginosa	ATCC 27853	-	-	-	-
Ps. aeruginosa	PEU3441	male	69	2019	urine
Ps. aeruginosa	PEU3440	female	46	2019	skin swab
Ent. faecalis	ATCC 51299	-	-	-	-
Ent. faecalis	PEU3443	male	24	2019	urine
Ent. faecalis	PEU3444	female	72	2019	wound swab
S. epidermidis	ATCC 12228	-	-	-	-
S. epidermidis	PEU3432	n.r.	n.r.	2019	n.r.
E. coli	ATCC 35218	-	-	-	-
E. coli	PEU3435	n.r.	n.r.	2019	n.r.
Str. pyogenes	PEU3430	male	3	2019	skin swab (ear)
Str. pyogenes	PEU3429	n.r.	n.r.	2019	n.r.

n.r. – not recorded

Table ST6: Bacteria strains II.

Species	Strain designation*	Gender	Age	Year	Source material	Subtype	Oxa
S. epidermidis	SE19_007	male	67	2019	central venous catheter tip	ST 820	S
S. epidermidis	SE19_008	female	92	2019	blood	ST 35	R
S. epidermidis	SE19_012	male	80	2019	blood	ST 2	R
S. epidermidis	SE19_014	female	55	2019	blood	ST 297	S
S. epidermidis	SE19_016	female	39	2019	tissue (spine)	ST 23	R
S. epidermidis	SE19_018	male	3	2019	cerebrospinal fluid	ST 495	S
S. epidermidis	SE19_021	male	59	2019	central venous catheter tip	ST 966	R
S. epidermidis	SE19_023	male	47	2019	surgery (tibia)	ST 2	R
S. epidermidis	SE19_025	female	81	2019	joint punctate (hip)	ST 19	S
S. epidermidis	SE19_026	male	51	2019	punctate (inguinal region)	ST 967	S
S. epidermidis	SE19_031	female	51	2019	abscess (neck)	ST 218	S
S. epidermidis	SE19_037	male	27	2019	tissue (clavicle)	ST 968	S
S. epidermidis	SE19_039	male	79	2019	tissue (ankle joint)	ST 2	R
S. epidermidis	SE19_040	female	84	2019	tissue (elbow joint)	ST 5	R
S. epidermidis	SE19_043	male	63	2019	tissue (knee)	ST 17	S
S. epidermidis	SE19_046	female	89	2019	joint punctate (knee)	ST 969	S
S. epidermidis	SE19_058	male	73	2019	punctate (lower leg)	ST 19	S
S. epidermidis	SE19_067	male	56	2019	tissue (clavicle)	ST 19	S
S. epidermidis	SE19_073	female	88	2019	implant (upper leg)	ST 970	S
S. epidermidis	SE19_088	male	83	2019	tissue (hip)	ST 87	R
S. epidermidis	SE19_097	female	68	2019	hip prosthesis (sonication)	ST 2	R
S. epidermidis	SE19_099	male	67	2019	cerebrospinal fluid	ST 595	S
S. aureus	MRSA1297	male	n.r.	2005	swab (nose)	ST 8	R
S. aureus	MRSA1337	male	n.r.	2005	urine catheter tip	ST 6327	R
S. aureus	MRSA1796	male	54	2009	blood	ST 398	R
S. aureus	MRSA2115	female	60	2010	blood	ST 5	R
S. aureus	MRSA2418	male	73	2012	blood	ST225	R
S. aureus	MRSA2516	male	73	2012	blood	ST 225	R
S. aureus	PEU3489	male	61	2020	wound swab (lower leg)	ST 30	S
S. aureus	PEU3490	male	71	2020	wound swab (hand)	ST 22	S
S. aureus	PEU3491	female	87	2020	blood (hip infection)	ST 15	S
S. aureus	PEU3492	female	78	2020	wound swab (forearm)	ST 1	S
S. aureus	PEU3493	male	60	2020	intraoperative swab (lower leg)	ST 8	S
S. aureus	PEU3494	female	52	2020	intraoperative swab (abscess leg)	ST 8	S
S. aureus	PEU3495	female	55	2020	intraoperative swab (foot)	ST 737	S
S. aureus	PEU3496	female	79	2020	blood	ST6328	S
S. aureus	PEU3497	female	97	2020	wound swab	ST 34	S
S. aureus	PEU3498	male	50	2020	tissue (lower leg)	ST 30	S
S. aureus	PEU3499	female	53	2020	tissue (finger)	ST 15	S
S. aureus	PEU3500	male	14	2020	skin swab (leg)	ST 1	S
S. aureus	PEU3501	male	59	2020	blood	ST 9	S
S. aureus	PEU3502	female	88	2020	wound swab (finger)	ST 30	S
S. aureus	PEU3503	male	4mts	2020	other (leg)	ST 188	S

n.r. – not recorded

* Uploaded to the database pubmlst.org and accessible via the hyperlink or by searching for the strain name/designation in this column as 'isolate' at the respective subpage at pubmlst.org.

References:

1. Wu, H. *et al.* Monitoring Plant Health with Near-Infrared Fluorescent H_2O_2 Nanosensors. *Nano Lett.* **20**, 2432–2442 (2020).
2. Selvaggio, G. *et al.* Exfoliated near infrared fluorescent silicate nanosheets for (bio)photonics. *Nat. Commun.* **11**, 1–11 (2020).
3. Nißler, R. *et al.* Quantification of the Number of Adsorbed DNA Molecules on Single-Walled Carbon Nanotubes. *J. Phys. Chem. C* **123**, 4837–4847 (2019).
4. Zheng, Y., Bachilo, S. M. & Weisman, R. B. Quenching of Single-Walled Carbon Nanotube Fluorescence by Dissolved Oxygen Reveals Selective Single-Stranded DNA Affinities. *J. Phys. Chem. Lett.* **8**, 1952–1955 (2017).
5. Bisker, G. *et al.* Protein-targeted corona phase molecular recognition. *Nat. Commun.* **7**, 1–14 (2016).
6. Bisker, G. *et al.* Insulin Detection Using a Corona Phase Molecular Recognition Site on Single-Walled Carbon Nanotubes. *ACS Sensors* **3**, 367–377 (2018).
7. Reich, S., Li, H., Garrity, O., Flavel, B. S. & Gordeev, G. Separation of Small-Diameter Single-Walled Carbon Nanotubes in One to Three Steps with Aqueous Two-Phase Extraction. *ACS Nano* **13**, 2567–2578 (2019).
8. Streit, J. K., Fagan, A. & Zheng, M. A Low Energy Route to DNA-Wrapped Carbon Nanotubes via Replacement of Bile Salt Surfactants. *Anal. Chem.* **89**, 10496–10503 (2017).
