# Supplementary material for: Remote near infrared identification of pathogens with multiplexed nanosensors
Source: Nat Commun. 2020 Nov 25;11:5995. doi: 10.1038/s41467-020-19718-5 (PMC7689463; doi:10.1038/s41467-020-19718-5)
Supplement: Supplementary file 3 — Reporting Summary [file 41467_2020_19718_MOESM3_ESM.pdf]

nature research

Corresponding author(s): Sebastian Kruss

Last updated by author(s): Oct 9, 2020

Reporting Summary

Nature Research wishes to improve the reproducibility of the work that we publish. This form provides structure for consistency and transparency in reporting. For further information on Nature Research policies, see our Editorial Policies and the Editorial Policy Checklist.

Statistics

For all statistical analyses, confirm that the following items are present in the figure legend, table legend, main text, or Methods section.

n/a	Confirmed	
☐	☒	The exact sample size (*n*) for each experimental group/condition, given as a discrete number and unit of measurement
☐	☒	A statement on whether measurements were taken from distinct samples or whether the same sample was measured repeatedly
☒	☐	The statistical test(s) used AND whether they are one- or two-sided<br>*Only common tests should be described solely by name; describe more complex techniques in the Methods section.*
☒	☐	A description of all covariates tested
☐	☒	A description of any assumptions or corrections, such as tests of normality and adjustment for multiple comparisons
☐	☒	A full description of the statistical parameters including central tendency (e.g. means) or other basic estimates (e.g. regression coefficient) AND variation (e.g. standard deviation) or associated estimates of uncertainty (e.g. confidence intervals)
☒	☐	For null hypothesis testing, the test statistic (e.g. *F*, *t*, *r*) with confidence intervals, effect sizes, degrees of freedom and *P* value noted<br>*Give P values as exact values whenever suitable.*
☒	☐	For Bayesian analysis, information on the choice of priors and Markov chain Monte Carlo settings
☐	☒	For hierarchical and complex designs, identification of the appropriate level for tests and full reporting of outcomes
☒	☐	Estimates of effect sizes (e.g. Cohen's *d*, Pearson's *r*), indicating how they were calculated

Our web collection on statistics for biologists contains articles on many of the points above.

Software and code

Policy information about availability of computer code

Data collection	Xeneth Software (version 2.7) (Xenics, Leuven Belgium) , Andor Solis Software (version 4.29.30012.0) (Andor Technology)
Data analysis	ImageJ (1.51k), GaphPad Prism (8.3), Origin (9.1), Python (3.7): matplotlib86 (version 3.0.3), NumPy87 (version 1.16.2), scikit-learn85 (version 0.20.3), Pandas (version 0.24.2) , R (3.6.1): ggbiplot (version 0.55)

For manuscripts utilizing custom algorithms or software that are central to the research but not yet described in published literature, software must be made available to editors and reviewers. We strongly encourage code deposition in a community repository (e.g. GitHub). See the Nature Research guidelines for submitting code & software for further information.

Data

Policy information about availability of data

All manuscripts must include a data availability statement. This statement should provide the following information, where applicable:

- Accession codes, unique identifiers, or web links for publicly available datasets
- A list of figures that have associated raw data
- A description of any restrictions on data availability

Additional characterization data on certain bacterial strains was made accessible online as indicated in the manuscript (https://pubmlst.org/bigsdb?db=pubmlst_sepidermidis_isolates&page=query). The Python code for stochastic sensor simulation is described in the manuscript and is available on request and online at https://gitlab.gwdg.de/m.dohmen/bacteria-sensing.git. The related source data files are available under 10.5281/zenodo.4072999.

Field-specific reporting

Please select the one below that is the best fit for your research. If you are not sure, read the appropriate sections before making your selection.

☒ Life sciences ☐ Behavioural & social sciences ☐ Ecological, evolutionary & environmental sciences

For a reference copy of the document with all sections, see nature.com/documents/nr-reporting-summary-flat.pdf

Life sciences study design

All studies must disclose on these points even when the disclosure is negative.

Sample size	No sample size calculation was performed. Chemical sensors were tested in a minimum of n = 3 independent experiments to determine mean and standard deviation (based on previous studies). For bacterial experiments one reference strain and >3 clinical isolates from different patients were used, as well as more than 20 clinical isolates each for S. aureus and S. epidermidis. This sample size was used to cover a range of technical and biological (epigenetic) variants within a bacterial strain, based on previous experiences on heterogeneity.
Data exclusions	No data was excluded from analysis
Replication	Control and repetitive experiments were performed as described in the manuscript (minimum of n = 3 independent experiments), whereby all attempts of replication were sucsessful.
Randomization	No randomization was performed, since the study's design wanted to distuinguish between known samples.
Blinding	No blinding was performed, since bacterially identified clinical isolates were analyzed in the study.

Reporting for specific materials, systems and methods

We require information from authors about some types of materials, experimental systems and methods used in many studies. Here, indicate whether each material, system or method listed is relevant to your study. If you are not sure if a list item applies to your research, read the appropriate section before selecting a response.

Materials & experimental systems

n/a	Involved in the study	
☒	☐	Antibodies
☒	☐	Eukaryotic cell lines
☒	☐	Palaeontology and archaeology
☒	☐	Animals and other organisms
☐	☒	Human research participants
☒	☐	Clinical data
☒	☐	Dual use research of concern

Methods

n/a	Involved in the study	
☒	☐	ChIP-seq
☒	☐	Flow cytometry
☒	☐	MRI-based neuroimaging

Human research participants

Policy information about studies involving human research participants

Population characteristics	Synovial liquid samples were acquired from patients with joint infections in the setting of routine treatment after obtaining informed consent. Data on participant/ patients demographics were not collected and were not associated to the bacterial strains found within the samples.
Recruitment	Consecutive patients with joint infection in the setting of routine diagnostics and treatment at a tertiary care center. No preselection in terms of bacterial infection type was done during sampling, which could biased the selection process. Therfore, we exclude any impact from the sample selection process to the results.
Ethics oversight	ethic proposal number 311/18, approved by medical faculty's ethic committee, University of Bonn

Note that full information on the approval of the study protocol must also be provided in the manuscript.
